# Supplementary figures and images for: Freshwater gobies 30 million years ago: New insights into character evolution and phylogenetic relationships of †Pirskeniidae (Gobioidei, Teleostei)
Source: PLoS One. 2020 Aug 24;15(8):e0237366. doi: 10.1371/journal.pone.0237366 (PMC7446829; doi:10.1371/journal.pone.0237366)

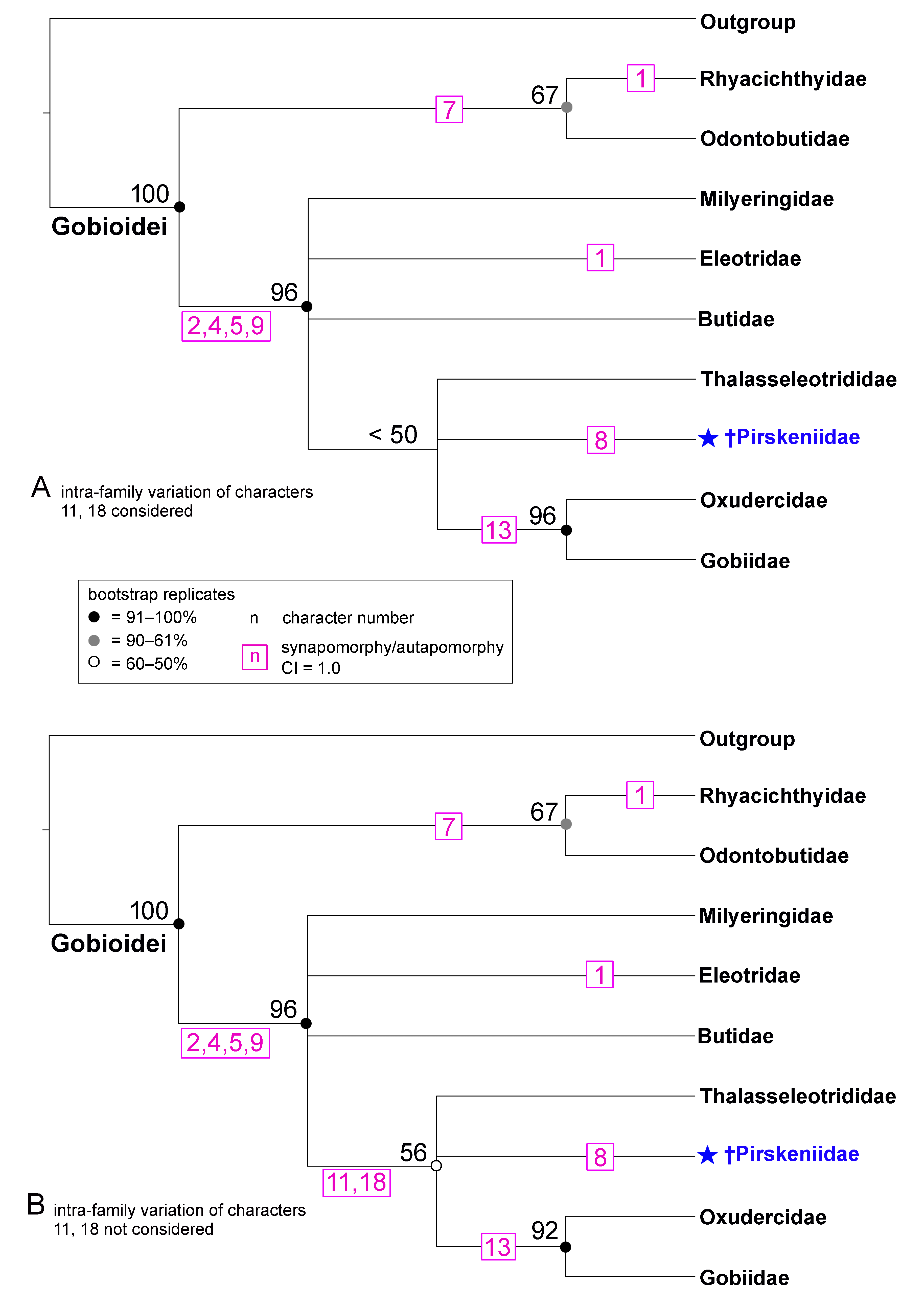

Supplement: S1 Fig — (A) 50% majority rule consensus tree based on 5 most parsimonious trees; matrix includes intra-family variation of characters 11 and 18 (see Table 2 for details). (B) 50% majority rule consensus tree based on 4 most parsimonious trees; matrix excludes intra-family variation of characters 11 and 18. For both trees, tree length = 33 steps, CI = 0.879, RI = 0.886. Tree search was conducted using a combination of ‘New Technology’ search options (parsimony ratchet, tree-drifting, tree-fusing) under equal weighting of characters. In the strict consensus tree of (A), the node leading to the Thalasseleotrididae and †Pirskeniidae had collapsed, and the two families were placed in a polytomy with Milyeringidae, Eleotridae and Butidae; the strict consensus tree of (B) had the same topology as the 50% majority rule consensus tree (trees not shown). Numbers in boxes are synapomorphies (respectively autapomorphies) as indicated by TNT; see Table 2 for character descriptions. Numbers at nodes are bootstrap percentages from 1000 pseudoreplicates. (TIF) [file pone.0237366.s001.tif]

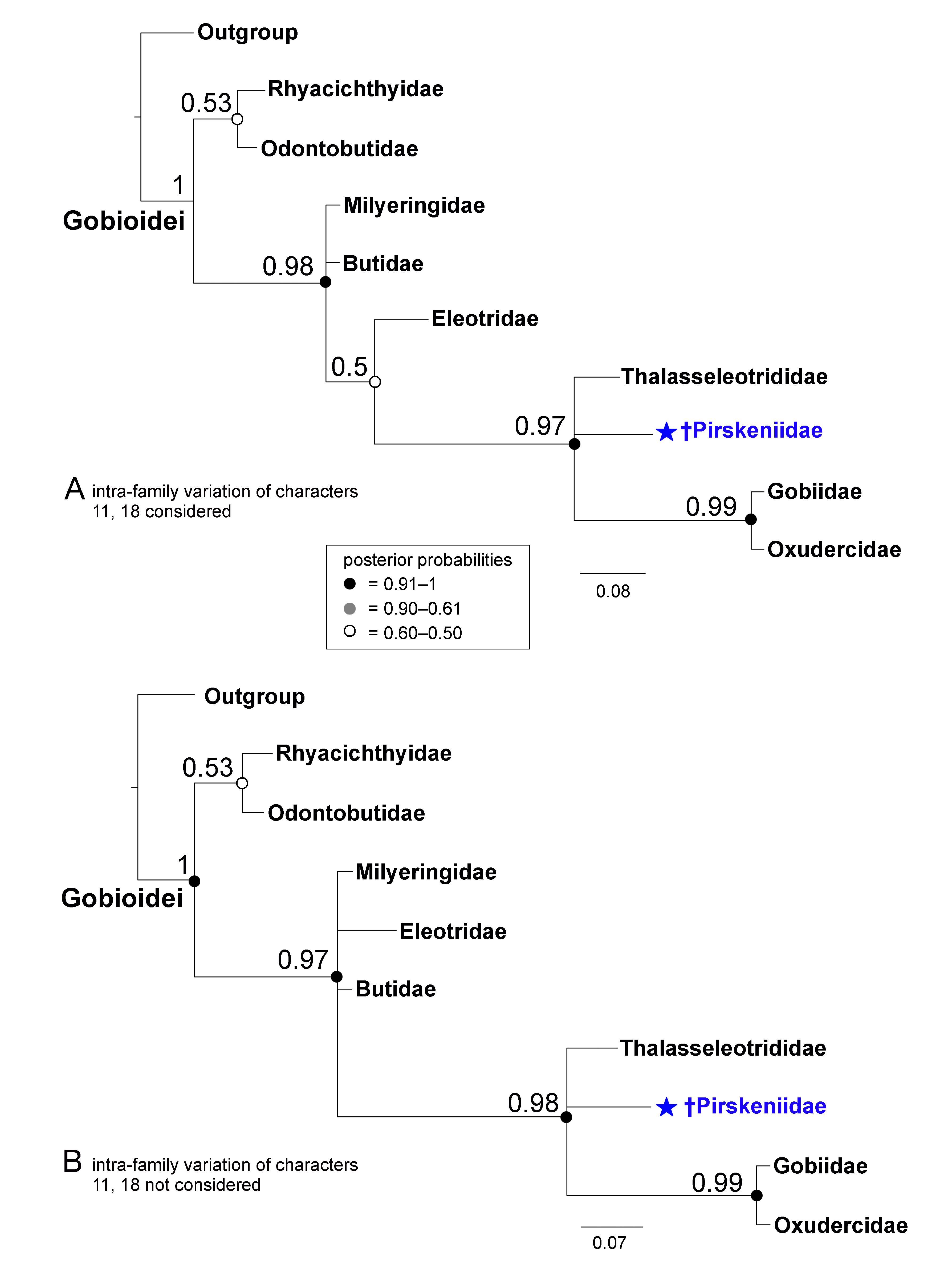

Supplement: S2 Fig — (A) 50% majority rule consensus tree based on 180,000 sampled trees from 2 independent runs; matrix includes intra-family variation of characters 11 and 18 (see Table 2 for details). (B) 50% majority rule consensus tree based on 180,000 sampled trees from 2 independent runs; matrix excludes intra-family variation of characters 11 and 18. For both trees the first 10% of trees were discarded as burn-in, chain length 106 generations, 1 cold and 3 heated chains per run, sampling every 10th generation. Numbers at nodes are posterior probabilities; scale bar indicates number of expected character changes per character. (TIF) [file pone.0237366.s002.tif]
