# Supplementary material for: Freshwater gobies 30 million years ago: New insights into character evolution and phylogenetic relationships of †Pirskeniidae (Gobioidei, Teleostei)
Source: PLoS One. 2020 Aug 24;15(8):e0237366. doi: 10.1371/journal.pone.0237366 (PMC7446829; doi:10.1371/journal.pone.0237366)
Supplement: Attachment — Submitted filename: PONE-D-20-14246, reviewed.pdf [file pone.0237366.s005.pdf]

Manuscript

Click here to access/download;Manuscript;Reichenbacher et al manuscript.docx

Freshwater gobies 30 million years ago: new insights into character evolution and phylogenetic relationships of †Pirskeniidae (Gobioidei, Teleostei)

Bettina Reichenbacher[1,2*], Tomáš Přikryl[3], Alexander F. Cerwenka[4], Philippe Keith[5], Christoph Gierl[1], Martin Dohrmann[6]

[1] Department of Earth and Environmental Sciences, Ludwig-Maximilians-Universität München, Richard-Wagner Straße 10, 80333 Munich, Germany; E-mails: b.reichenbacher@lrz.uni-muenchen.de, c.gierl@lrz.uni-muenchen.de

[2] GeoBio-Center LMU, Ludwig-Maximilians-Universität München, Munich, Germany

[3] Institute of Geology of the Czech Academy of Sciences, Rozvojová 269, CZ-165 00 Praha, Czech Republic; E-mail: prikryl@gli.cas.cz

[4] SNSB-Bavarian State Collection of Zoology, Section Evertebrata varia, 81247, Munich, Germany; E-mail: cerwenka@snsb.de

[5] Laboratoire de Biologie des organismes et écosystèmes aquatiques (BOREA), Muséum national d'Histoire naturelle, CNRS, IRD, SU, Paris, France; E-mail: philippe.keith@mnhn.fr

[6] SNSB-Bavarian State Collection of Palaeontology and Geology, Richard-Wagner Straße 10, 80333 Munich, Germany; E-mail: dohrmann@snsb.de

Corresponding author: E-mail: b.reichenbacher@lrz.uni-muenchen.de

Abstract

The modern Gobioidei (Teleostei) comprise eight families, but the extinct †Pirskeniidae from the lower Oligocene of the Czech Republic indicate that family diversity was greater in the past. However, the validity of the †Pirskeniidae has been questioned and its single genus †*Pirskenius* has been assigned to the extant family Eleotridae in previous works. The objective of this study is to clarify the status of the †Pirskeniidae. Whether or not the †Pirskeniidae should be synonymised with the Eleotridae is also interesting from a biogeographical point of view as Eleotridae are not present in Europe or the Mediterranean Sea today. We present new specimens and re-examine the material on which the two known species of †*Pirskenius* are based (†*P. diatomaceus* Obrhelová, 1961; †*P. radoni* Přikryl, 2014). To provide a context for phylogenetically informative characters related to the palatine and the branchiostegal rays, an early-branching gobioid (*Rhyacichthys*), an eleotrid (*Eleotris*) and a gobiid (*Gobius*) were subjected to micro-CT analysis. The new data justify revalidation of the family †Pirskeniidae, and a revised diagnosis is presented for both †*Pirskenius* and †Pirskeniidae. Phylogenetic analyses indicate a possible placement of †Pirskeniidae as sister to the Thalasseleotrididae + Gobiidae + Oxudercidae, or as sister to the Thalasseleotrididae. Considering the fossil record, the conquest of freshwater habitats by gobioids in the early Oligocene apparently had generated new lineages that finally were not successful and became extinct shortly after they had diverged. Moreover, the current data on fossil gobioids sugge[illegible] that Eleotridae were never part of the European ichthyofauna.

Introduction

Living gobioids are distributed worldwide and constitute one of the most species-rich vertebrate suborders, with approximately 2,200 species belonging to > 270 genera [1]. They are small, mostly benthic fishes that form a significant faunal component of reefs and other

shallow marine ecosystems, and are also abundant in brackish and freshwater habitats [2]. Among the extant Gobioidei, eight families have been recognised based on morphological characteristics and molecular phylogenetics, i.e. the Rhyacichthyidae, Odontobutidae, Milyeringidae, Eleotridae, Butidae, Thalasseleotrididae, Gobiidae, and Oxudercidae; Rhyacichthyidae and Odontobutidae are sister to all other gobioid families (Fig 1) [3-7]. Gobiidae and Oxudercidae represent the most derived clade (Fig 1). ~~They~~ share three derived characters that are in principle recognisable in fossils, namely the possession of five branchiostegal rays (vs. six in the other groups), a 'T-shaped' (vs. an 'L-shaped') palatine bone, and united pelvic fins [4, 8-10].

Fig 1. Phylogeny of the Gobioidei according to Betancur et al. [11], Nelson et al. [1], and Thacker [6].

6brG = gobioid families with six branchiostegal rays, 5brG = gobioid families with five branchiostegal rays. Modified after Gierl and Reichenbacher [12].

Although the first appearance of a lineage in the fossil record is always younger than the primary divergence event, the fossil record can provide crucial data on the lineage's distribution and diversification in the past. The oldest gobioid species known from clearly freshwater environments are based on skeletons dated to the early Oligocene [13-16]. They are represented by four species, †*Pirskenius diatomaceus* Obrhelová, 1961, †*P. radoni* Přikryl, 2014, '*Gobius' gracilis* Laube, 1901 (which probably represents an extinct genus) and †*Lepidocottus papyraceus* (Agassiz, 1832). While †*L. papyraceus* is a member of the Butidae [15, 17], the family relationships of the others are either unknown ('*Gobius' gracilis*) or disputed (†*Pirskenius*). The original description of †*Pirskenius* by Obrhelová [14] considered it to represent a new, extinct family, †Pirskeniidae Obrhelová, 1961. But later it was

suggested that †Pirskeniidae cannot be sustained and that its single known genus †*Pirskenius* should be assigned to the Eleotridae [16, 18]. In that case, †*Pirskenius* would represent the oldest skeleton-based species of the family Eleotridae. In addition, it would indicate that eleotrids had conquered European inland waters by the early Oligocene.

This study set out to resolve the status of the †Pirskeniidae and thus contribute to a better understanding of the ancient diversity and biogeography of gobioid families. To achieve this, previously available †*Pirskenius* material and new finds were investigated in the light of a comparative micro-CT study of the branchiostegal rays and the palatine in an early-branching gobioid (*Rhyacichthys*), an eleotrid (*Eleotris*) and a gobiid (*Gobius*).

Geological setting

The type locality of †*Pirskenius diatomaceus* Obrhelová, 1961 is Knížecí, in the north of the Czech Republic, and the type locality of †*P. radoni* is Byňov, which lies about 60 km SW of Knížecí (Fig 2). Both sites are located in the volcanoclastic complex of the České Středohoří Mountains. During the Oligocene, this region experienced significant tectonic and volcanic activity, which also gave rise to the emergence of several freshwater lakes, as indicated by the fossil finds at Knížecí, Byňov and several other sites (Fig 2) [13, 16, 18]. Knížecí is a mine dump on the northern slope of the Hrazený hill (whose former name, 'Pirskensberg', accounts for the designation of the genus), east of the village of Knížecí, near Šluknov [14, 19]. The ancient outcrop was an approximately 5-m-thick succession of diatomites and sandy-to-coaly clays, overlain by tephritic rocks (Kopecký in [20]). The outcrop itself must have been re-filled or destroyed shortly after its initial description, because all fossils were collected from diatomites associated with the mine dump (Zlatko Kvaček, pers. comm., [14]). The fish fossils from Knížecí were made up solely of the gobioid †*P. diatomaceus* and the cyprinid

†*Protothymallus elongatus* (Kramberger in Gorjanović-Kramberger, 1885) [13, 14, 18, 21]. In addition, well-preserved plants have been reported [20, 22]. Obrhelová [14] assumed †*P. diatomaceus* to be an Oligo-Miocene species because, at the time of her study, the age of the Knížecí deposit was not clear. Bellon et al. [19] provided an ^{40}K-^{40}Ar age of 29.5±1.5 MYA (= early Oligocene) based on 'one surface sample taken at about 510 m in height'. As the original outcrop no longer existed at that time, this sample was probably collected from the mine dump.

Fig 2. Map of the area with the type localities (stars) of †*Pirkenius diatomaceus* and †*P. radoni* and other fossiliferous sites (F).
All sites are located in the České Středohoří Mountains (northern Czech Republic). Redrawn after Gaudant [18].

Byňov, the type locality of †*P. radoni*, is also a mine dump, situated to the east of the village of Byňov [16]. Apart from four specimens of †*P. radoni*, several plant fossils have been described [23]. Their composition is similar to that of the floral assemblages from Seifhennersdorf and Kundratice (see Kvaček in [16]), and these sites are early Oligocene in age [13]. Accordingly, both Byňov and Knížecí can now be attributed to the early Oligocene.

Institutional abbreviations

MNHN, Muséum national d'Histoire naturelle, Paris, France; NMP, National Museum, Prague, Czech Republic; SNSB-ZSM, Zoological State Collection, Munich, Germany.

Materials and methods

Fossil specimens

Three well preserved articulated fossil skeletons of †*Pirskenius* (collection numbers NMP Pv 11669, 11671, 11672) were newly available from Knížecí. They had been collected by Ervín Knobloch in 1958 and 1961, but were only recently discovered in the 'Knobloch collection' of the National Museum in Prague (see Kvaček et al. [22]). Further fossil specimens comprised 44 skeletons of the original material of †*P. diatomaceus* described by Obrhelová (1961) (collection numbers NMP PC 2770–PC 2822) and the four known specimens of †*P. radoni* described by Přikryl [16] (collection numbers MT PA1480–1484).

The definition of morphometric characters follows Liu et al. [24]. Morphometric measurements of †*P. diatomaceus* were taken with ImageJ v1.51a 64-bit [25] based on the digital images. Measurements of †*P. radoni* were adopted from Přikryl [16]. Measurements were standardised based on the standard length of the fossil; only in the case of the eye diameter standardization was based on the head length. Osteological and meristic characters were examined with a stereomicroscope (Leica MZ6, Leica M165 FC, Olympus SZX 12) or a Keyence digital microscope, each equipped with a digital camera (Canon EOS 1000D, Leica DFC 450, Olympus DP72, Keyence VH-Z20 UR). Topographic terms used to designate bones refer to their natural anatomical position, even if the bone was displaced in the fossil specimen. Counts of vertebrae include the terminal centrum, counts of rays in the second dorsal fin and anal fin include every discernible ray.

Comparative material

Specimens of representative extant species of the families Rhyacichthyidae, Eleotridae and Gobiidae were used for comparative studies of the branchiostegal rays and the shape of the palatine head: (1) *Rhyacichthys guilberti* Dingerkus and Séret, 1992, Rhyacichthyidae (two specimens, Malekula island, Vanuatu, Oceania, MNHN 2019-0113); (2) *Eleotris pisonis* (Gmelin, 1789), Eleotridae, type species of ~~the name-giving genus~~ *Eleotris* Bloch and

Schneider, 1801 (two specimens, ZSM 9393 from Curaçao Island, Caribbean Sea, ZSM 41704 from Rio Cerere, Costa Rica); (3) *Gobius incognitus* Kovačić and Šanda, 2016, Gobiidae (one specimen, Pelješac peninsula, Croatia, NMP 6V 146150).

To examine their branchiostegal rays and the palatine, scans were taken with a phoenix nanotom m µ-CT-scanner equipped with a cone-beam scanner (GE Sensing and Inspection Technologies GmbH, Wunstorf, Germany). Fish specimens were mounted in a plastic vessel and stabilised with paper wipes. A small amount of 75% ethanol was added to the vessel to avoid drying. Specimens were scanned over 360° with three averaged images per rotation position, using a tungsten ('standard') target. Further details of each scan are given in Table 1.

Table 1. Technical details of the micro-CTs of extant gobioids.

Subsequently, all datasets were reconstructed separately with the software phoenix datos|x 2 (GE Sensoring and Inspection Technologies GmbH, Germany), cropped and converted to 8bit using VGStudio MAX 2.2 (Volume Graphics, Heidelberg, Germany). Co-registering and merging of the multiscans and visualization were done manually using Amira 6.4 software (FEI Visualization Sciences Group, Burlington, MA, USA).

Phylogenetic analysis

Derived states of morphological characters according to Hoese and Gill [4], Gill and Mooi [9] and the results of the present study (Table 2) were mapped on a recently published molecular phylogeny of the Gobioidei provided by Thacker et al. [7]. Plausible positions of the †Pirskeniidae (see Systematic Palaeontology section for justification) were determined as suggested by the character state distributions.

Table 2. Morphological characters for classification of Gobioidei.

Characters that refer to soft tissue or delicate bony structures and thus are unlikely to be preserved in a fossil are indicated in italics. Characters 21–25 refer to autapomorphies.

In addition, a cladistic analysis was conducted using the morphological data matrix presented by Hoese and Gill [4]. This matrix contained 16 osteological characters, 10 of which can principally be preserved in fossils. We discarded three characters (preopercular mandibular canal; infraorbital canal; interhyal position) as their phylogenetic information solely concerned the Rhyacichthyidae and was exactly the same as two other characters (penultimate branchiostegal ray position, lateral line), which – in contrast to the three discarded characters – can be easily recognized in fossils. In addition, the binary character 15 (branchiostegal ray number) of Hoese and Gill [4] was transformed into a new multistate character termed 'serial number of expanded last branchiostegal ray' (Table 2, character 12) because it is not simply the number of branchiostegal rays that is important, but also the fact that the last branchiostegal ray is expanded (see [26]). The other character states were the same as used by Hoese and Gill [4], except for characters 1 (adductor mandibulae tendon attachment) and 8 (penultimate branchiostegal ray position), which were modified from binary to multistate characters taking into account the osteological details described (but not coded) by the authors. To this list of characters, the palatine shape with a new definition of its character states (Table 2, character 14), and the synapomorphies recognized by Gill and Mooi [9] for the Thalasseleotrididae + Gobiidae/Oxudercidae and Gobiidae/Oxudercidae, respectively, were added (Table 2, characters 15–20). The entire list of characters comprises 20 phylogenetically informative characters.

The taxon set of Hoese and Gill [4] included the families Rhyacichthyidae, Odontobutidae, Butidae (as Butinae), Eleotridae (as Eleotridinae) and Gobiidae/Oxudercidae (as Gobiinae) (see Hoese and Gill [4]: tables 1, 2 and 'Osteological Material Examined' for included species; no specific outgroup had been defined). We adopted this taxon set, with the following modifications: the families Thalasseleotrididae and Milyeringidae (subsumed by Hoese and Gill [4] with the 'Eleotridinae' and 'Butinae', respectively) were added, and a generalized percomorph was included as outgroup. †Pirskeniidae (see Systematic Palaeontology section for justification) was incorporated into the data matrix by inserting character states for nine characters (for details see Table 2). The newly compiled matrix was edited in Mesquite 3.61 [27]. Phylogenetic analysis was performed under maximum parsimony in TNT 1.5 (Willi Hennig Society Edition; [28]), using a combination of ‘New Technology’ search options, i.e. parsimony ratchet, tree-drifting and tree-fusing. Characters were treated as unordered and equally weighted. Clade support was assessed using standard bootstrapping [29], 1000 replicates and absolute frequencies values. Phylogenetic trees were visualized and edited in FigTree 1.4.4 [30].

Anatomical abbreviations

A, anal fin; aa, angulo-articular; abd vert, abdominal vertebrae; AP, anal fin pterygiophores inserting before haemal spine of first caudal vertebra; bh, basihyal; BH, maximal depth at onset of D1; BL, body length (horizontal line from first vertebral centrum to end of hypural plates); br, branchiostegal rays; C, principal caudal fin rays; caud vert, caudal vertebrae; ce, ceratohyal; ce (a), anterior ceratohyal; ce (p), posterior ceratohyal; cl, cleithrum; CPH, caudal peduncle height (maximal depth of caudal peduncle); CPL, caudal peduncle length (horizontal line from end of anal fin to end of hypural plates); D1, first dorsal fin; D1 pt-form, pterygiophore formula of D1; D2, second dorsal fin; D2C, distance between end of D2 and

first dorsal procurrent ray of caudal fin; den, dentary; ED, eye diameter (horizontally); ect, ectopterygoid; ent, entopterygoid; ethm, ethmoid; ethm pr pal, ethmoid process of palatine; fr, frontal; HL, head length; hm, hyomandibular; LPect, maximum length pectoral fin rays; LPelv, maximum length pelvic fin rays; mx, maxilla; mxl pr pal, maxillary process of palatine; orb, orbit; op, opercle; pal, palatine; Pect, pectoral fin; Pelv, pelvic fin; pmx, premaxilla; pop, preopercle; prC, procurrent rays of caudal fin; psph, parasphenoid; ptt, posttemporal; r, radials; SL, standard length; SN/A, distance from tip of the snout to begin of A; SN/D1, distance from tip of the snout to begin of D1; SN/D2, distance from tip of the snout to begin of D2; sop, subopercle; sy, symplectic; vert, vertebrae; vo, vomer.

Results

The palatine bone of the extant species

The position of the palatine bone within the skull and its terminology are shown in Figure 3. In *Rhyacichthys guilberti*, the palatine head displays a very long maxillary process and a much smaller, angular ethmoid process (Figs 4A2 and 4B2). The palatine of *Eleotris pisonis* is L-shaped (*sensu* Regan [10]), i.e. showing a blunt, clearly discernible maxillary process, but no ethmoid process (Figs 3A3 and 4C2). In contrast, the palatine of *Gobius incognitus* is 'T-shaped' (*sensu* [10]), i.e. the palatine head has two prominent processes, one for connection to the maxillary and the other one for articulation with the ethmoid (Figs 3B3 and 4D2).

Fig 3. Anatomical position and details of the palatine (yellow) of extant gobioid species. (A) *Eleotris pisonis* (ZSM 9393), skull in lateral (A1) and dorsal (A2) views, left palatine (A3) in lateral view. (B) *Gobius incognitus* (NMP6V 146150), skull in lateral (B1) and dorsal (B2) views, left palatine (B3) in lateral view. All images based on μ-CT scanning.

Abbreviations: ethm, ethmoid; ethm pr pal, ethmoid process of palatine; mx, maxilla; mxl pr pal, maxillary process of palatine; pal, palatine; pmx, premaxilla.

Fig 4. Anatomical position and details of the palatine, the ceratohyal bone, and the branchiostegal rays of extant gobioid species.

(A, B) *Rhyacichthys guilberti* (MNHN 2019-0113a, b), skull (A1, B1), left palatine (A2, B2), left ceratohyal (A3, B3) with five and six branchiostegal rays, respectively, right ceratohyal (A4, B4) with six branchiostegal rays. (C) *Eleotris pisonis* (ZSM 9393), skull (C1), left palatine (C2), left ceratohyal (C3) with six branchiostegal rays. (D) *Gobius incognitus* (NMP6V 146150), skull (D1), left palatine (D2), left ceratohyal (D3) with five branchiostegal rays. All images based on µ-CT scanning, all images show lateral view.

The branchiostegal rays of the extant species

One of the specimens of *Rhyacichthys guilberti* examined displays six branchiostegal rays at each hyoid bar (Figs 4B3 and 4B4). The second individual has six branchiostegal rays on the right (Fig 4A4), but only five on the left hyoid bar (Fig 4A3). The specimens of *Eleotris pisonis* display six branchiostegal rays (Fig 4C3), while the specimen of *Gobius incognitus* reveal a number of five branchiostegal rays (Fig 4D3). In all studied specimens, the last branchiostegal ray is considerably expanded compared to the preceding ones and ~~is~~ articulate~~d~~s with the posterior ceratohyal ~~bone~~ (Fig 4). In *R. guilberti* the penultimate branchiostegal ray is also associated with the posterior ceratohyal (Figs 4A3, 4A4, 4B3, 4B4), while in *E. pisonis* (Fig 4C3) and *G. incognitus* (Fig 4D3) the penultimate branchiostegal ray is 'shifted' anteriorly and articulates with the anterior ceratohyal.

Systematic palaeontology

Teleostei Müller, 1845 *sensu* Arratia [31]

Percomorphaceae *sensu* Betancur-R. et al. {Betancur-R., 2013 #4416}

Gobiiformes *sensu* Betancur-R. et al. {Betancur-R., 2017 #4484}

Suborder Gobioidei Günther, 1880

Family †Pirskeniidae Obrhelová, 1961

Genus †*Pirskenius* Obrhelová, 1961

†*Pirskenius diatomaceus* Obrhelová, 1961

Figs 5–7, Tables 3 and 4

1961 *Pirskenius diatomaceus* n. sp. – Obrhelová, p. 111, pl. I–XV, text-figs 1–29.

2014 *Pirskenius diatomaceus* Obrhelová, 1961. – Přikryl: p. 188.

Material. New specimens NMP Pv 11669, 11671, 11672, holotype NMP PC 2769 (no. 8 in {Obrhelová, 1961 #251}) and specimens NMP PC 2770–PC 2822.

Provenance and age. Knížecí (Czech Republic); early Oligocene.

Preservation. The holotype is a moderately well preserved imprint of a complete individual. Many of the further specimens are well preserved (some as part and counterpart), but most specimens are incomplete, lacking either the caudal or the anterior part of the body. The three new specimens (Pv 11669, 11671, 11672) are very well preserved and revealed several previously unknown characters of †*P. diatomaceus* (see below).

Fig 5. Fossil skeletons of †*Pirskenius diatomaceus* Obrhelová, 1961.

(A) Specimen Pv 11669, skull (ventrolateral view) and anteriormost portion of the body (lateral view) exhibiting seven branchiostegal rays. (B) Specimen Pv 11672, complete skeleton with slightly disarticulated skull. (C) Specimen Pv 11671, skeleton with well-preserved dorsal fins, anal fin and caudal peduncle. (D) Specimen PC 2799, almost complete skeleton. Abbreviations: br, branchiostegal rays; op, opercle; pop, preopercle.

General description. The total length of †*P. diatomaceus* ranges between 13 and 60 mm. The head and the eyes are relatively large (HL 24% SL, EL 27–37% HL, Table 3; Figs 5A, 5C, 6A, 7A, 7C1). The oral gape is terminal and opened (Fig 7A). The body is long and slender, tapering only slightly towards the caudal fin (BL 75.4–76.7% SL, BH 9–13% SL; Figs 5B–D, 7C1). The paired fins are relatively short (13–14% SL), with the pectoral fin being slightly longer than the pelvic fin. The second dorsal and the anal fin are placed approximately midway along the body, with the anal fin inserting slightly behind the second dorsal fin (Figs 5B, 5C, 7A, 7C1). The bases of the second dorsal and the anal fin are relatively short (13–15% SL and 10–15% SL, respectively). The caudal peduncle is long and slender (CPL 24.6–33.7% SL, CPH 7.7–11.6% SL), and appears to be relatively longer in the largest specimens (Table 3). The caudal fin is fan-shaped to slightly rounded in form (Fig 5B).

Fig 6. Osteology of the skull of †*Pirskenius diatomaceus* Obrhelová, 1961.
(A) Specimen Pv 11669 (ventrolateral view) exposing bones of right (r) and left (l) side, with palatine (pal), ectopterygoid (ect) and entopterygoid (ent) preserved in anatomical connection (upper arrow indicates ethmoid process of palatine, lower arrow points to ventral tip of palatine). (B) Specimen PC 2786 showing branchiostegal rays 1–6 articulating with anterior ceratohyal, and expanded branchiostegal ray 7 associated to posterior ceratohyal. Abbreviations: aa, angulo-articular; br, branchiostegal rays; ce, ceratohyal; ce (a), anterior ceratohyal; ce (p), posterior ceratohyal; cl, cleithrum; den, dentary; ect, ectopterygoid; ent, entopterygoid; fr, frontal; hm, hyomandibular; mx, maxilla; op, opercle; pal, palatine; pmx, premaxilla; psph, parasphenoid; sop, subopercle; sy, symplectic; vo, vomer.

Table 3. Morphometric data of †*Pirskenius diatomaceus*.

Specimens are arranged according to their standard length (SL). Values depict measurements in mm, values in brackets refer to % SL, only in case of eye diameter (ED) to % of head length (HL). * indicates that SL was calculated based on measurement of body length (BL) or HL or caudal peduncle length (CPL) by assuming a proportion of BL = 76% of SL, HL = 24% of SL, and CPL = 32% SL, respectively, based on measurements of complete specimens. For abbreviations see Material and methods.

Skull (Figs 5A and 6). The frontal bones show the typical gobioid condition, with a trapezoid shape, a broad postorbital section and a slender interorbital segment (Fig 6A). The width of the interorbital segment is about 21–25% of the width of the postorbital sector (PC 2786: 21.3%, PC 2787: 22.9%, PC 2791: 24.8%, Pv 11669: c. 25%); the value of 16% provided for specimen PC 2786 by Obrhelová ([14]: table 14, as no. 22) could not be confirmed. The parasphenoid is a long, slender bone, broadened only in the posterior region of the neurocranium; anteriorly it is associated with a broad, triangular vomer, which does not bear teeth (PC 2791, Pv 11669; Fig 6A). The bones of the ethmoid and otic regions and of the occiput are not clearly discernible. Parietal bones and infraorbital bones are absent.

The jaw joint is located slightly in front of (PC 2787, PC 2804), or below the anteriormost portion of the orbit (PC 2707, PC 2772). The premaxilla has a relatively long ascending process and a somewhat shorter articular process. The horizontal ramus of the premaxilla bears a prominent, wedge-shaped postmaxillary process, which gradually tapers posteriorly, so that the posterior end of the premaxilla is slender (Fig 6A, also visible in specimen PC 2786). The premaxilla bears small conical teeth that are arranged in several rows (well visible in specimen PC 2772). The maxilla is a slender, slightly curved bone (Fig 6A), its articular process is usually obscured by other bones or not well preserved; according

to Obrhelová [14], it is relatively long and forms a U-shaped articular facet for the articulation with the palatine. The dentary is relatively slender, and posteriorly bifurcated (Figs 6A and 7A). The relatively large head of Pv 11669 reveals details of the lower jaw dentition: its left dentary is preserved in labial view and comprises two different sectors (Fig 6A), the anterior sector bears irregularly arranged clusters of teeth (sandpaper type), while the teeth in the posterior sector are organised in rows.

The palatine is a robust, straight, and relatively short bone, whose posteroventral tip ends approximately midway along the ectopterygoid (indicated by the lower arrow in Fig 6A). According to Obrhelová [14], the maxillary process of the palatine is straight and slender, continuing in the direction of the longitudinal axis of the palatine, but it is actually not visible in any of the specimens examined because other bones conceal it. Some specimens show the respective part of the palatine to be associated with the lateral side of the vomer, and it seems probable that Obrhelová [14] mistakenly identified the thickened anterolateral margin of the vomer as a long slender maxillary process of the palatine. Notably, the palatine head bears a short lateral ethmoid process (indicated by the upper arrow in Fig 6A). The ectopterygoid is elongate, approximately as long as the maxilla, and widens towards the quadrate (Fig 6A). The entopterygoid appears to be as long as the ectopterygoid and is straight (Fig 6A). The quadrate has a triangular body with a distinct articular head; anteriorly, it is firmly attached to the ventral margin of the ectopterygoid, posteriorly it has a long process that extends to the preopercle. The symplectic is a relatively robust rod that articulates with the posterior margin of the corpus quadrati, i.e. to the fossa quadrati (Fig 6A). A broad gap between the symplectic and the preopercle (suspensorium fenestra or symplectical foramen) is clearly discernible (Fig 6B). The slender metapterygoid is not connected to the quadrate. The hyomandibular is a relatively broad and somewhat rounded bone, with three distinguishable processes (Fig 6B).

The hyoid bar consists of a relatively long anterior ceratohyal bone, which begins as a slender element and then broadens, and a relatively short, broad and triangular posterior ceratohyal bone (Fig 6B); a small gap is visible between the two parts and could represent an originally cartilaginous region. The number of branchiostegal rays is always seven (Figs 5A and 6B, clearly detectable also in PC 2774, PC 2775, PC 2791, PC 2798). The three anteriormost branchiostegal rays articulate with the slender portion of the anterior ceratohyal, and are thin and regularly spaced (Fig 6B). After a gap follow the relatively thick and closely spaced branchiostegal rays 4–6, which are attached to the widened portion of the anterior ceratohyal. The sixth branchiostegal ray is located just before the gap between the anterior and posterior ceratohyal (Fig 6B). The seventh branchiostegal ray is the broadest of the series and articulates with the posterior ceratohyal (Fig 6B). The opercular bones are best preserved in specimens Pv 11669 (Fig 5A) and PC 2786 (Fig 6B). The subopercle is elongate and crescent-shaped, the opercle is triangular, and the preopercle has a slender lower and an upper arm of almost equal length. Specimen Pv 11669 reveals a well preserved bony preopercular canal (*sensu* Hoese and Gill [4]) extending along the horizontal and vertical branch of the preopercle (Fig 5A).

Fig 7. Fossil skeletons of †*Pirskenius diatomaceus* Obrhelová, 1961 showing details of fins.

(A) Specimen PC 2772 exhibiting dorsal and anal fins (posterior part not preserved). (B) Counterpart of (A) (PC 2773) displaying radials (r1–r4) and remains of the pectoral fin (pect) and first dorsal fin with D1 pterygiophore formula 3(122110) (pterygiophores indicated by arrows). (C1–3) Specimen PC 2791, overview (C1), close-up of caudal fin with well-preserved procurrent rays (C2), close-up of separated pelvic fins (C3). Abbreviations: bh, basihyal; ptt, posttemporal.

Table 4. Meristic data of †*Pirskenius diatomaceus*.

Specimens are arranged according to their collection numbers. For abbreviations see Material and methods.

Vertebral column (Fig 7B, Table 4). The number of abdominal vertebrae is 11, and there are 16–17 caudal vertebrae, with the exception of specimen PC 2791, which has 18. The neural spines are slender and of approximately equal length all along the vertebral column. Haemal spines are as long as neural spines, apart from that of the first caudal vertebra, which exhibits a somewhat shortened haemal spine, and preural vertebra 2, which has an enlarged haemal spine. The abdominal vertebrae have robust parapophyses, in some specimens their connection with the ribs is preserved. The total number of rib pairs is 8–9, beginning at vertebra 3 and extending to vertebra 10 or 11; the first seven rib pairs are robust and relatively long, the posteriormost pairs are thinner and shorter (Fig 7B, also well visible in PC 2799, Pv 11669, Pv 11671). Epipleurals are associated with the first four vertebrae (Pv 11669). There are no supraneurals.

Caudal skeleton (Figs 5B and 7C2, Table 4). The caudal skeleton is composed of two wide hypural plates (composed of hypurals 1+2 and 3+4), the fifth hypural plate is small and thin. A robust parhypural and a large, single epural are present. The caudal fin is fan-shaped. It usually comprises 13, rarely 14 principal rays, of which seven are placed in the dorsal section. The number of procurrent caudal rays is up to 14 dorsally, and up to 11 ventrally.

Unpaired fins (Figs 5B, 5C, 7A, 7B, Table 4). The first dorsal fin (D1) has six or seven spines, the last of which is set apart from the preceding spine by a small gap. The length of

the spines decreases continuously from the first or second spine to the last. Each D1 spine is supported by a long, slender pterygiophore (indicated by the arrows in Fig 7B); only the first pterygiophore is broadened distally. The first pterygiophore inserts behind the neural spine of vertebra 3 and the last pterygiophore behind the neural spine of vertebra 6 or 7 (depending on whether six or seven D1-spines are developed). Between the last D1 pterygiophore and the first D2 pterygiophore is an interneural space without a pterygiophore. The D1 pterygiophore formula is recognisable in four specimens, it is 3(122110) in the case of seven D1 spines, and 3(12210) in the case of six spines.

The second dorsal fin (D2) begins slightly in front of the insertion of the anal fin. The first element is a slender spine, which is slightly shorter (i.e. about four fifths) than the following ray. The total number of rays is nine to ten. All rays are segmented and branched; the anterior rays are long, the more posterior rays become increasingly shorter. The first D2 pterygiophore is a slender bone and probably bipartite, i.e. comprising a long proximal and a relatively shorter distal radial, but no middle radial (specimen PC 2772).

The anal fin inserts beneath the first or second ray of D2. It consists of a slender spine, which is somewhat shorter than the following nine to ten rays. The number of anal fin pterygiophores that insert anterior to the haemal spine of the first caudal vertebra is two (specimens PC 2773, Pv 11672) or three (PC 2791).

Paired fins and their skeleton (Fig 7A, B, C1, C3, Tables 2, 3). The cleithrum is long, with a slender dorsal part and a widened ventral portion. The supracleithrum is robust and associated with a bifurcated posttemporal, the dorsal process of which is longer than the ventral. There is no postcleithrum and no scapula. The pectoral fins insert on the lower one-third of the flank and are relatively short (c. 14% SL). Each fin is supported by four elongate radials with ovate-shaped gaps in between (also visible in PC 2785/2786). According to

Obrhelová [14], the number of pectoral fin rays is 15–16, rarely 12, 14, or 17 rays. However, a number of 12–14 pectoral fin rays is displayed by specimens PC 2804, PC 2791 and Pv11671 and appears more feasible. The pelvic girdle is a robust and medially fused bone; its anterior part is slightly rounded to heart-shaped, the posterior portion pointed and triangular. The pelvic fins are inserted just under or slightly behind the pectoral fins and are clearly separated. Each pelvic fin includes one spine and five rays. The rays are slightly shorter (12.6% SL) than those of the pectoral fin, and clearly terminate anterior to the anal fin.

Scales (Fig 7A, B). Ctenoid scales cover the flanks. Because they overlap, only their somewhat thickened posterior margins are visible; circuli and radii are not preserved. A single row of relatively long and slender ctenii is present at the posterior scale margin. Scale width can be estimated to be 1.5–1.7% SL (measured below the second dorsal fin and close to the vertebral column). Transverse rows include 5–6 scales, longitudinal rows about 60. Up to eight cycloid scales on the hypural plates are recognisable in several specimens. The head is scale-less. The lateral line is absent.

†*Pirskenius radoni* Přikryl, 2014

2014 *Pirskenius radoni* n. sp.; Přikryl: 189, Figs 1–4.

General description. †*Pirskenius radoni* has similar body proportions as †*P. diatomaceus*, except that its head is slightly larger and the body is less elongate (Table 5). The scales of this species are not preserved. A re-examination of the holotype (MT PA1480) and paratypes (MT PA1482, 1483) revealed some additional details, which are reported in the following. For a complete and detailed description see Přikryl [16].

Table 5. Comparison of morphometric data (in % of SL) and meristic counts between the two species of †*Pirskenius*.

Ranges and counts for †*P. diatomaceus* refer to the values provided in Tables 3 and 4, data for †*P. radoni* originates from Přikryl [16] and this study. For abbreviations see Material and methods.

Skull. The preserved remains of the premaxilla suggest the presence of a relatively high postmaxillary process, as in *P. diatomaceus*. The shape of the ectopterygoid resembles a strongly elongated triangle (rather than the 'L' shape depicted in Přikryl [16]). The endopterygoid is stick-like and located dorsally to the ectopterygoid. The palatine extends along half the length of the ectopterygoid, as in †*P. diatomaceus*. Přikryl [16] noted a clear maxillary process of the palatine and concluded that the palatine is L-shaped. However, the anterior part of the palatine is not clearly readable. Preserved fragments of skeletal tissue suggest the presence of the maxillary process, but whether the ethmoid process was developed is not clear, because the vomer covers the area in which it would be expected. Since a short ethmoid process is present in †*P. diatomaceus,* and the palatine configuration is unlikely to vary between congeneric species, we assume that a short ethmoid process was also present on the palatine of †*P. radoni*; thus the palatine of †*P. radoni* was not exactly L-shaped.

The anterior ceratohyal bone shows a slightly concave dorsal margin and a strongly concave ventral margin; its anterior part is relatively narrow and it becomes about twice as deep in the posterior section. Seven branchiostegal rays are present. In size, shape, and configuration, they resemble those of †*P. diatomaceus* (see above).

Caudal skeleton. The caudal skeleton of †*P. radoni* is composed of the same elements as in †*P. diatomaceus* (see Přikryl [16]). A single epural with a clearly discernible suture is present,

suggesting that two epural bones were fused. Compared to †*P. diatomaceus*, the caudal fin of †*P. radoni* is more rounded (see Přikryl [16]). It is composed of 14 principal rays (eight dorsally and six ventrally, not 7 + 7 as reported by Přikryl [16]). Several dislocated procurrent rays are recognisable dorsally and ventrally, probably more than five in each case.

Unpaired fins. The first dorsal fin is composed of seven spines. The most probable D1 pterygiophore formula is 4(32110), as the first pterygiophore seems to be located posterior to the neural spine of the fourth vertebra (rather than after the third, as noted in the original description). The first D1 spine is shorter than the succeeding ones, apart from the seventh spine, which is the shortest. As in †*P. diatomaceus*, the seventh spine is separated from the preceding ones by a small gap. The second dorsal fin is composed of one spine and eight rays. The spine has more or less the same length as the adjacent rays, which become shorter posteriorly. The anal fin is composed of a single spine and nine rays. The spine is approximately as long as the anteriormost ray, the subsequent rays become gradually smaller posteriorly. The number of anal fin pterygiophores is four.

Paired fins and their skeleton. The pectoral girdle, which is only partially preserved, includes an arch-shaped cleithrum (that is somewhat straighter in its ventral portion), which is connected to the posterior part of the skull via the supracleithrum and a V-shaped posttemporal (note that the interpretative drawing in Přikryl [16]: Fig 4 incorrectly depicts the latter elements as fused). The ventral limb of the posttemporal is significantly shorter than the dorsal. The ventral part of the coracoid is more or less discernable.

Discussion

†*Pirskenius* in light of the new data

Obrhelová [14] introduced the family †Pirskeniidae with the single genus †*Pirskenius*. She provided a detailed diagnosis of both family and genus, together with a very comprehensive description of †*P. diatomaceus*, the sole species of †*Pirskenius* known at that time. Her work is excellent in many respects, especially when one considers that very little was known about the osteology of gobioids when she began her study. Nevertheless, there are some inconsistencies and obscurities in her work, some of which were previously noted by Springer [32], Nelson [33], Gaudant [18] and Přikryl [16]. These concern mainly the (1) number of branchiostegal rays, (2) presence or absence of entopterygoid, (3) presence of ctenoid or cycloid scales, (4) number of abdominal vertebrae, and (5) terminology of the caudal skeleton. In the present study, these issues could be resolved.

1) In the original diagnosis of †Pirskeniidae and †*Pirskenius,* a number of six to seven branchiostegal rays is mentioned, but the species description refers exclusively to seven rays. Here, we confirm the presence of seven branchiostegal rays in all specimens of †*P. diatomaceus* and †*P. radoni* examined.
2) The entopterygoid is reported as 'present or absent' in the original family diagnosis and species description, but a 'very narrow' entopterygoid is mentioned in the original genus diagnosis. Whether this bone is present or absent is of systematic relevance at the level of family [4, 34]. Thus, 'presence or absence' of this bone in a given taxon is unlikely. According to the results of our study and Přikryl [16], an entopterygoid is present in both †*P. diatomaceus* and †*P. radoni.*
3) The scales are reported as 'ctenoid or cycloid' in the family diagnosis, but as 'ctenoid' in the genus diagnosis. The re-investigation of †*P. diatomaceus* revealed the presence of ctenoid scales on the body, but cycloid scales occur on the base of the caudal fin (above the hypural plates).
4) The number of abdominal vertebrae was reported as '11 (12)' in the genus

diagnosis of †*Pirskenius*. In contrast, a number of exclusively 11 was mentioned in the species description, which is also consistent with the results of this study. Twelve abdominal vertebrae are found only in †*P. radoni* ([16] this study).

5) The terminology in use for the caudal skeleton at the time Obrhelová [14] conducted her study differs from that employed today [32, 35]. Judging from her drawings and photos of the caudal skeleton of †*P. diatomaceus* in Obrhelová [14] and the results of this study, it is clear that her 'two upper Epiuralia' correspond to hypural plate 5 and the epural, and that her 'lower Epiuralia' represents the parhypural. This is the common condition of the caudal skeleton in gobioids [36, 37].

Moreover, some previously unknown characters of †*Pirskenius* could be discerned in the present study, mostly based on the new specimens of ~~†*P. diatomaceus*~~. These relate to the postmaxillary process of the premaxilla, the teeth on the dentary, the shape of the palatine head, the bony preopercle canal support, the articulation of the sixth branchiostegal ray, and the presence of an interneural gap between the first and second dorsal fin (see above). In addition, the D1 pterygiophore formula, which was first introduced by Birdsong [38], could be established. Unfortunately, it sheds no light on the taxonomic position of †*Pirskenius*. Of the two formulas detected for †*P. diatomaceus*, the formula 3(12210) commonly occurs in the Oxudercidae, occasionally in some eleotrids, and (as an exception) in the butid *Kribia* (see [39]), whereas the formula 3(122110) is only known from some eleotrids (*Philypnodon*, *Dormitator*, *Gobiomorphus*), the odontobutid *Perccottus* and (as an exception), from the gobiid *Psilotris* (see [39]). The formula 4(32110) (†*P. radoni*) is unknown among extant gobioids ([39] and unpublished data of BR).

Hereafter, a revised diagnosis of the genus †*Pirskenius* is presented. It excludes several of the characters provided in the original genus diagnosis because, according to the

current knowledge of the osteology of gobioids, they appear in many groups. These characters relate to the anteriorly elongate and posteriorly broadened parasphenoid; the toothed premaxillary and dentary; the presence of small, curved, conical oral teeth; the symplectic foramen; the opercle without spines; the bifurcated posttemporal; the presence of four radials; the presence of vertebral centra that are longer than high; the caudal skeleton with two large hypural plates (Hy), comprising Hy1+2 and Hy3+4, and a small hypural plate 5; and the presence of branched fin rays (see [4-6, 32, 36, 37, 40-44]). The shape of the frontal bones is also excluded, as it has been shown to differ between congeneric species [12]. A non-toothed vomer, although occurring in many groups of gobioids, is not excluded because one species of the Eleotridae, *Eleotris vomerodentata* Maugé, 1984, endemic to Madagascar, was described as new because of plenty teeth on the vomer [45].

Revised diagnosis of †*Pirskenius* Obrhelová, 1961

Small fish, with the following unique combination of characters: up to 6 cm total length, with typical gobioid placement of the median and paired fins and long caudal peduncle (25–34% of SL); head relatively large (HL 23–32% SL); non-toothed vomer; premaxilla with distinct postmaxillary process; dentary relatively slender; entopterygoid present; palatine with short lateral ethmoid process (maxillary process not visible); seven branchiostegal rays; postcleithrum absent, scapula absent; separated pelvic fins; 11–12 abdominal vertebrae; total number of vertebrae 27–28, rarely 29; well-developed parapophyses; pectoral fin rays 12–14; D1 VI–VII; D2 I8–10; A I9–10; two to four anal fin pterygiophores inserting anterior to haemal spine of first caudal vertebra; 13–14 principal caudal fin rays; single epural.

The family †Pirskeniidae – valid or not?

Gaudant [18] and Přikryl [16] had suggested that the combination of characters presented as

diagnostic for the †Pirskeniidae is not unique among the Gobioidei. Both authors considered the palatine of †*Pirskenius* to have an eleotrid shape (*sensu* Regan [10]) and synonymised †Pirskeniidae with the Eleotridae.

However, a number of seven branchiostegal rays, as in †*Pirskenius*, is unknown among extant Gobioidei [40]. This number occurs in their next close relatives, the Trichonotidae, Apogonidae and Kurtidae [46-48], but the members of these families lack the expansion of the last branchiostegal ray as is characteristic for the Gobioidei [26]. Přikryl [16] regarded the presence of seven branchiostegal rays in †*Pirskenius* as an ancestral state of the Eleotridae, because Akihito [8] had reported one specimen of *Odontobutis obscurus* (Temminck and Schlegel, 1845) as showing the same condition. However, this observation refers to an apparently anomalous character state, as the other 11 specimens examined by Akihito had six branchiostegals. The comparative material used here also includes one instance of a similar irregularity: in one of the specimens of *Rhyacichthys guilberti* the left hyoid bar has five branchiostegal rays (Fig 4A3), instead of the normal condition of six rays. As *Rhyacichthys* represents an early-branching gobioid [4, 5], and as five branchiostegal rays is the condition of the modern Gobiidae and Oxudercidae [9], this finding also can be treated as an exception. Occasional deviations in the number of branchiostegal rays can thus occur as random cases, without phylogenetic significance. As a consequence, the presence of seven branchiostegal rays sets †Pirskeniidae apart from all extant gobioid families and represents an autapomorphy for this family.

The second crucial character of †*Pirskenius* is the occurrence of a palatine head with a short lateral ethmoid process (Fig 6a). Probably †*Pirskenius* did not have a truly T-shaped palatine (*sensu* Regan [10]) as is characteristic for the Gobiidae and Oxudercidae [34, 49]. However, the condition of a palatine that is not exactly L-shaped is derived as it is not present

in the Odontobutidae, Eleotridae and Butidae [8, 34, 50]. †Pirskeniidae exhibits further derived characters (see above and Table 2) and it is thus justified to resurrect the family.

Revised diagnosis of †Pirskeniidae Obrhelová, 1961

Small fish, up to 6 cm total length, with seven branchiostegal rays and the typical gobioid placement of the median and paired fins. Entopterygoid present; palatine head with short lateral ethmoid process (maxillary process not visible); uppermost radial of pectoral fin adjoins cleithrum; scapula absent; postcleithrum absent; separated pelvic fins; ctenoid scales without transforming ctenii.

Phylogenetic position of †Pirskeniidae

To analyse the possible phylogenetic position of the †Pirskeniidae we used two approaches: (i) derived states of morphological characters (see Table 2) were plotted on the molecular tree of the Gobioidei of Thacker et al. [7] and the †Pirskeniidae were added to this tree manually as suggested by the character state distributions; (ii) a phylogenetic analysis was performed under maximum parsimony in TNT 1.5 using the character matrix described in the Methods section (Table 2, characters 1–20).

The first approach suggests two options to place †Pirskeniidae within the molecular tree of the Gobioidei provided by Thacker et al. [7]: †Pirskeniidae is sister to all Gobioidei, except Rhyacichthyidae and Odontobutidae (Fig 8A), or †Pirskeniidae is sister to Thalasseleotrididae + Gobiidae + Oxudercidae (Fig 8B). In the first option, particular weight is laid on the penultimate branchiostegal ray position (see states of character 8 in Table 2). The plesiomorphic condition is exhibited by the Rhyacichthyidae, in which the penultimate branchiostegal ray is placed at the posterior ceratohyal (= state 0; see Figs 4A3-4 and 4B3-4). In †Pirskeniidae the penultimate branchiostegal ray articulates at the anterior ceratohyal, but

near to the gap that separates this part of the bone from the posterior ceratohyal (= state 1, s[illegible] Fig 6B). Such a condition is also known for the Odontobutidae [4], which could indicate a placement of †Pirskeniidae close to the Odontobutidae. Eleotridae, Butidae and Gobiidae are more specialised insofar as the penultimate ray articulates with the anterior ceratohyal, clearly anterior to the gap to the posterior ceratohyal (= state 2; see Fig 4C3, D3). However, †Pirskeniidae reveals two further characters that preclude a placement near the Odontobutidae, namely an uppermost radial that adjoins the cleithrum, while the scapula is absent (see states of character 4 in Table 2), and presence of ctenoid scales that lack transforming ctenii (see states of character 7 in Table 2). These two characters support monophyly of all gobioids (including †Pirskeniidae) except Rhyacichthyidae and Odontobutidae (see [4] and Figure 8). If we consider the exact position of the penultimate branchiostegal as phylogenetically more informative than other characters (see below), then †Pirskeniidae would be set apart from the remaining families and represent a stem lineage of all gobioids except Rhyacichthyidae and Odontobutidae, as indicated in Figure 8A.

Fig 8. Two possible phylogenetic positions of the †Pirskeniidae based on mapping of morphological characters on a recently published molecular tree of extant Gobioidei. (A) †Pirskeniidae is sister to all extant gobioid families except Rhyacichthyidae and Odontobutidae. (B) †Pirskeniidae is sister to Thalasseleotrididae + Gobiidae + Oxudercidae. Synapomorphies or autapomorphies are indicated with pink bars, convergently derived character states are shown in light blue, derived pterygiophore formulas are depicted with yellow bars, multistate characters are followed by a slash (/) after which the character state is indicated. For character numbers see Table 2. Tree adapted from Thacker et al. [7] 'based on DNA sequences of ten nuclear protein coding genes with a taxon sampling expanded from Near et al. [51]'.

The second option of the same approach uses character 1 (position of penultimate branchiostegal ray) as it was defined in the original publication by Hoese and Gill [4], i.e. the derived condition is that the penultimate branchiostegal ray articulates with the anterior ceratohyal, irrespective at which position. In this case, †Pirskeniidae displays the derived condition and does not split off early as shown in Figure 8A. Option 2 is further supported because †Pirskeniidae displays a palatine that is not exactly L-shaped and lacks the dorsal postcleithrum (see states of characters 14 and 18 in Table 2). The palatine shape would be unique for †Pirskeniidae and the clade Thalasseleotrididae + Oxudercidae + Gobiidae, and the absent dorsal postcleithrum would remain a synapomorphy in the precise definition of Gill and Mooi [9] (Fig 8B). In the alternative scenario of option 1 (Fig 8A) characters 14 and 18 would both require convergent evolution in †Pirskeniidae and Thalasseleotrididae + Oxudercidae + Gobiidae. ~~It thus seems that~~ option 2, with Thalasseleotrididae + Oxudercidae + Gobiidae being sister to †Pirskeniidae ~~more reliably reflects~~ the phylogenetic relationships of the ~~†Pirskeniidae~~.

The second approach, maximum parsimony analysis of the extant gobioid families and the †Pirskeniidae based on 20 phylogenetically informative morphological characters, yielded a single most parsimonious tree with a length of 31 and a relatively low degree of homoplasy (consistency index = 0.903; retention index = 0.921). Some important relationships are recognisable (Fig 9). Although Rhyacichthyidae and Odontobutidae are not sister groups, they occur as outgroups to all other Gobioidei, which is consistent with the cladistic analysis of Hoese and Gill [4] based on 16 morphological characters, and molecular phylogenies (e.g. [3, 7]). Thalasseleotrididae is closer to Gobiidae + Oxudercidae, which form a well supported clade, than to the other extant families, also consistent with molecular phylogenies. Notably, †Pirskeniidae appears closely related to these three families (albeit with low bootstrap

support), consistent with option 2 above. More precisely, it is reconstructed as the sister group of Thalasseleotrididae, but this needs further investigation as bootstrap support for this relationship is < 50% and no clear synapomorphies of these two families are apparent to us (see Fig 9).

Fig 9. Phylogenetic position of the †Pirskeniidae based on cladistic analysis. Single most-parsimonious tree based on 20 phylogenetically informative morphological characters using TNT. Tree length = 31 steps, consistency index = 0.903, retention index = 0.921. Numbers in boxes are synapomorphies (respectively autapomorphies for †Pirskeniidae) as produced by TNT; see Table 2 for character descriptions. Numbers at nodes are bootstrap percentages from 1000 pseudoreplicates (values <50% not shown).

Concluding remarks

Here, we have established that †*Pirskenius diatomaceus* and †*P. radoni* represent species of the extinct family †Pirskeniidae, and are not members of the extant family Eleotridae. Previously reported fossil remains of putative eleotrids from the Oligocene and lower Miocene of Europe all refer to isolated bones [13, 18] or otoliths [52-54]. As isolated bones do not allow one to differentiate between Eleotridae and Butidae (see Fig 8), and as the 'eleotrid' otoliths actually represent otoliths of the Butidae [17], it appears that the Eleotridae actually were not present in Europe in the past. This implies that the Eleotridae and Butidae, both ~~distributed~~ nowadays in Africa, Madagascar + adjacent islands, India, Micronesia, Polynesia, and Melanesia, had different biogeographic histories. In the Oligocene and early Miocene, Butidae were a common element of inland water bodies in Europe and along the

Mediterranean littoral [15, 17], but Eleotridae were probably absent from this area at that time.

†Pirskeniidae, which was restricted to the early Oligocene (c. 29–30 Ma) of Central Europe, is phylogenetically close to the extant clade Thalasseleotrididae + Gobiidae + Oxudercidae. This indicates that the Thalasseleotrididae, today limited to New Zealand and temperate Australia [9], possibly was more widespread in the past. Furthermore, most of the members of the Thalasseleotrididae + Gobiidae + Oxudercidae are marine, whereas †*Pirskenius* was limited to freshwater (or slightly saline) inland lakes. This is consistent with the notion of Thacker [6] that the sister clades of the Gobiidae + Oxudercidae – the Thalasseleotrididae was not yet recognized at the time of her study – are mostly freshwater and brackish water inhabitants.

Moreover, the existence of the †Pirskeniidae suggests that the Gobioidei were more diverse (as indicated by the number of families) in the Oligocene than they are today. Judging from the fossil record, it seems that gobioids did not begin to conquer freshwater environments prior to the Oligocene (30 Ma). Since then, the adaptations to the new habitats may well have produced lineages that were ultimately unsuccessful and became extinct within a short time after their divergence.

Acknowledgements

We are grateful to Zlatko Kvaček (Charles University, Prague, Czech Republic) for providing the new specimens of †*Pirskenius*. We thank B. Ekrt (National Museum, Prague, Czech Republic) and M. Radoň (Museum of Teplice, Czech Republic) for access to the specimens in their care and for the opportunity to use the Keyence microscope at the Palaeontological Department of the National Museum in Prague. We also thank U. Schliewen and D. Neumann

(both SNSB-ZSM, Munich, Germany) for providing access to specimens kept in the SNSB-ZSM collection, and B. Ruthensteiner (SNSB-ZSM) for technical support concerning visualization of micro-CT scans. We thank J. Vukić (Charles University, Prague, Czech Republic) and R. Šanda (National Museum, Prague, Czech Republic) for personally delivering the specimen of *Gobius ignotus*. We are indebted to H. Larson (Museum and Art Gallery of the Northern Territory, Darwin, Australia) and P. Chakrabarty (LSU Museum of Natural Science, Baton Rouge, Louisiana, USA) for providing information on the number of branchiostegal rays in *Milyeringa*. We thank the Vanuatu Environment Unit for granting access to *Rhyacichthys* (Permit Numbers ENV326/001/1/07/DK and ENV326/001/1/08/D). We acknowledge funding for this project from the Deutsche Forschungsgemeinschaft to B.R. (RE-1113/20). T.P.'s research was institutionally supported by the Czech Academy of the Sciences, Institute of Geology (RVO67985831). The Willi Hennig Society is acknowledged for making TNT available free of charge.

References

1. Nelson JS, Grande TC, Wilson MVH. Fishes of the World, Fifth Edition. Hoboken, New Jersey: John Wiley & Sons, inc.; 2016. 752 p.
2. Patzner RA, Van Tassell JL, Kovačić M, Kapoor BG, editors. The biology of gobies. 1 ed. Enfield, New Hampshire: Science Publishers Inc.; 2011.
3. Agorreta A, San Mauro D, Schliewen U, Van Tassell JL, Kovačić M, Zardoya R, et al. Molecular phylogenetics of Gobioidei and phylogenetic placement of European gobies. Molecular phylogenetics and evolution. 2013;69(3):619–33. doi: 10.1016/j.ympev.2013.07.017. PubMed PMID: MEDLINE:23911892.
4. Hoese DF, Gill AC. Phylogenetic relationships of eleotridid fishes (Perciformes, Gobioidei). B Mar Sci. 1993;52(1):415–40. PubMed PMID: ISI:A1993KX23000015.

5. Miller PJ. The osteology and adaptive features of *Rhyacichthys aspro* (Teleostei: Gobioidei) and the classification of gobioid fishes. Journal of Zoology. 1973;171(3):397–434. doi: 10.1111/j.1469-7998.1973.tb05347.x.

6. Thacker CE. Phylogeny of Gobioidei and placement within Acanthomorpha, with a new classification and investigation of diversification and character evolution. Copeia. 2009;2009(1):93–104. doi: 10.1643/Ci-08-004. PubMed PMID: ISI:000263748000013.

7. Thacker CE, Satoh TP, Katayama E, Harrington RC, Eytan RI, Near TJ. Molecular phylogeny of Percomorpha resolves *Trichonotus* as the sister lineage to Gobioidei (Teleostei: Gobiiformes) and confirms the polyphyly of Trachinoidei. Molecular phylogenetics and evolution. 2015;93:172–9. Epub Aug. doi: 10.1016/j.ympev.2015.08.001. PubMed PMID: MEDLINE:26265255; PubMed Central PMCID: PMC26265255.

8. Akihito. A systematic examination of the gobiid fishes based on the Mesopterygoid, Postcleithra, Branchiostegals, pelvic fins, Scapula, and Suborbital. Jpn J Ichthyol. 1969;16(3):93–104. Epub Jun 28, 2010. doi: 10.11369/jji1950.16.93. PubMed PMID: BCI:BCI197051058231.

9. Gill AC, Mooi RD. Thalasseleotrididae, new family of marine gobioid fishes from New Zealand and temperate Australia, with a revised definition of its sister taxon, the Gobiidae (Teleostei: Acanthomorpha). Zootaxa. 2012;3266(1):41–52. doi: 10.11646/zootaxa.3266.1.3.

10. Regan CT. The osteology and classification of the gobioid fishes. The Annals and Magazine of Natural History [Eighth Series]. 1911;8(48):729–33.

11. Betancur-R. R, Wiley EO, Arratia G, Acero A, Bailly N, Miya M, et al. Phylogenetic classification of bony fishes. BMC evolutionary biology. 2017;17(1):162. doi: 10.1186/s12862-017-0958-3. PubMed PMID: 28683774.

12. Gierl C, Reichenbacher B. Revision of so-called *Pomatoschistus* (Gobiiformes, Teleostei) from the late Eocene and early Oligocene. Palaeontologia Electronica. 2017;20.2.33A:1–17. doi: 10.26879/721. PubMed PMID: WOS:000405188500014.

13. Böhme M. Revision of the cyprinids from the Early Oligocene of the České Středohoři Mountains, and the phylogenetic relationships of *Protothymallus* Laube 1901 (Teleostei,

Cyprinidae, Gobioninae). Acta Musei Nationalis Pragae, Series B - Historia Naturalis. 2007;63(2–4):175–94. PubMed PMID: BCI200900046183.

14. Obrhelová N. Vergleichende Osteologie der tertiären Süsswasserfische Böhmens (Gobioidei). Sbornik Paläont. 1961;26:103–92.

15. Pandolfi L, Carnevale G, Costeur L, Del Favero L, Fornasiero M, Ghezzo E, et al. Reassessing the earliest Oligocene vertebrate assemblage of Monteviale (Vicenza, Italy). Journal of Systematic Palaeontology. 2017;15(2):83–127. Epub Mar 16, 2016. doi: 10.1080/14772019.2016.1147170.

16. Přikryl T. A new species of the sleeper goby (Gobioidei, Eleotridae) from the České Středohoří Mountains (Czech Republic, Oligocene) and analysis of the validity of the family Pirskeniidae. Paläontol Z. 2014;88(2):187–96. Epub Jun 28, 2013. doi: 10.1007/s12542-013-0188-y.

17. Gierl C, Reichenbacher B, Gaudant J, Erpenbeck D, Pharisat A. An extraordinary gobioid fish fossil from southern France. PloS one. 2013;8(5):e64117. Epub May 15. doi: 10.1371/journal.pone.0064117. PubMed PMID: 23691158; PubMed Central PMCID: PMCPMC3655028.

18. Gaudant J. Note complémentaire sur l'ichthyofaune oligocène de Seifhennersdorf (Saxe, Allemagne) et de Varnsdorf, Kundratice, Lbín, Skalice, Knížecí, etc. (Bohême, République tchèque). Annalen des Naturhistorischen Museums in Wien - Serie A (Mineralogie und Petrographie, Geologie und Paläontologie, Archäozoologie, Anthropologie und Prähistorie). 2009;111:281–312. PubMed PMID: BCI200900349724.

19. Bellon H, Bůžek Č, Gaudant J, Kvaček Z, Walther H. The České Středohoří magmatic complex in Northern Bohemia 40K-40Ar ages for volcanism and biostratigraphy of the Cenozoic freshwater formations. Newsl Stratigr. 1998;36(2–3):77–103. doi: 10.1127/nos/36/1998/77. PubMed PMID: WOS:000075588000002.

20. Knobloch E. Die oberoligozäne Flora des Pirskenberges bei Šluknov in Nord-Böhmen. Sbor Ústř Úst geol, odd paleont. 1961;26:241–315.

21. Obrhelová N. Die Karpfenfische des Tchechoslovakischen Süßwassertertiär. Časopis pro mineralogii a geologii. 1969;14(1):39–52.

22. Kvaček Z, Teodoridis V, Zajícová J. Revision of the Early Oligocene Flora of Hrazený Hill (formerly Pirskenberg) In Knížecí near Šluknov, North Bohemia. Sbornik Národního Muzea v Praze Rada B Prirodni Vedy. 2015;71(1-2):55-102. doi: 10.14446/amnp.2015.55. PubMed PMID: BCI:BCI201600833911.

23. Radoň M. Nové paleontologické nálezy v terciérních intravulkanických sedimentech České středohoří. Geoscience Research Reports. 2006;39:94–7.

24. Liu HTH, Ahnelt H, Balma GAC, Delmastro GB. First record of *Gobius roulei* (Gobiidae) in the Ligurian Sea. Cybium. 2009;33(3):253–4. PubMed PMID: WOS:000272599200014.

25. Schneider CA, Rasband WS, Eliceiri KW. NIH Image to ImageJ: 25 years of image analysis. Nat Methods. 2012;9(7):671–5. Epub 2012/08/30. doi: 10.1038/nmeth.2089. PubMed PMID: WOS:000305942200020.

26. McAllister DE. Evolution of the branchiostegals and classification of teleostome fishes. Bull natn Mus Can (Biol Ser). 1968;77:1–239.

27. Maddison WP, Maddison DR. Mesquite: a modular system for evolutionary analysis. Version 3.6 http://mesquiteproject.org. 3.6 ed2019.

28. Goloboff PA, Catalano SA. TNT version 1.5, including a full implementation of phylogenetic morphometrics. Cladistics. 2016;32(3):221–38. Epub Apr 25. doi: 10.1111/cla.12160. PubMed PMID: WOS:000376269000001.

29. Felsenstein J. Confidence Limits on Phylogenies: An Approach Using the Bootstrap. Evolution. 1985;39(4):783–91. doi: 10.2307/2408678. PubMed PMID: WOS:A1985APJ8100007.

30. Rambaut A. FigTree. Tree Figure Drawing Tool version 1.4.4. http://tree.bio.ed.ac.uk/software/figtree/. 1.4.4 ed. Edinburgh: University of Edinburgh; 2018.

31. Arratia G. The monophyly of Teleostei and stem-group teleosts. Consensus and disagreements. In: Arratia G, Schultze H-P, editors. Mesozoic Fishes 2 - Systematics and Fossil Record. München: Verlag Dr. Friedrich Pfeil; 1999. p. 265–334.

32. Springer VG. *Tyson belos*, new genus and species of Western Pacific fish (Gobiidae, Xenisthminae), with discussions of gobioid osteology and classification. Washington, D. C.: Smithsonian Institution Press; 1983. 40 p.

33. Nelson JS. Fishes of the World, Fourth edition. fourth ed. Hoboken, New Jersey: John Wiley & Sons, Inc.; 2006. 624 p.

34. Hoese DF. Gobioidei: relationships. In: Moser HG, Richards WJ, Cohen DM, Fahay MP, Kendall J, A. W., Richardson SL, editors. Ontogeny and systematics of fishes. Gainesville, Florida: American Society of Ichthyologists and Herpetologists; 1984. p. 588–91.

35. Schultze H-P, Arratia G. The caudal skeleton of basal teleosts, its conventions, and some of its major evolutionary novelties in a temporal dimension. In: Arratia G, Schultze H-P, Wilson MVH, editors. Mesozoic Fishes 5 – Global Diversity and Evolution. 5. München: Verlag Dr. Friedrich Pfeil; 2013. p. 187–246.

36. Tornabene L, Deis B, Erdmann MV. Evaluating the phylogenetic position of the goby genus *Kelloggella* (Teleostei: Gobiidae), with notes on osteology of the genus and description of a new species from Niue in the South Central Pacific Ocean. Zool J Linn Soc. 2018;183(1):143–62. Epub Dec 16, 2017. doi: 10.1093/zoolinnean/zlx060. PubMed PMID: WOS:000432303500006.

37. Wiley EO, Johnson GD. A teleost classification based on monophyletic groups. In: Nelson JS, Schultze H-P, Wilson MVH, editors. Origin and phylogenetic interrelationships of teleosts. München: Verlag Dr. Friedrich Pfeil; 2010. p. 123–82.

38. Birdsong RS. The osteology of *Microgobius signatus* Poey (Pisces: Gobiidae), with comments on other gobiid fishes. Bull Florida State Mus, Biol Sci. 1975;19(3):135–87.

39. Birdsong RS, Murdy EO, Pezold FL. A study of the vertebral column and median fin osteology in gobioid fishes with comments on gobioid relationships. B Mar Sci. 1988;42(2):174–214. PubMed PMID: ISI:A1988N503000003.

40. Larson HK, Murdy EO, Gill AC. Suborder Gobioidei. In: Carpenter KE, Niem VH, editors. FAO species identification guide for fishery purposes The living marine resources of the Western Central Pacific Volume 6 Bony fishes part 4 (Labridae to Latimeriidae), estuarine crocodiles, sea turtles, sea snakes and marine mammals. 6. Rome: FAO; 2001. p. 3574–609.

41. Lord C, Bellec L, Dettaï A, Bonillo C, Keith P. Does your lip stick? Evolutionary aspects of the mouth morphology of the Indo-Pacific clinging goby of the *Sicyopterus* genus (Teleostei:

Gobioidei: Sicydiinae) based on mitogenome phylogeny. Journal of Zoological Systematics and Evolutionary Research. 2019;57(4):910–25. Epub May 30. doi: 10.1111/jzs.12291.

42. Mennesson MI. Taxonomie, phylogenie et dispersion du genre *Eleotris* (Teleostei: Gobioidei: Eleotridae) dans l'Indo-pacifique. [PhD dissertation]. Paris2016.

43. Pezold F. Phylogenetic analysis of the genus *Gobionellus* (Teleostei: Gobiidae). Copeia. 2004;2004(2):260–80. doi: 10.1643/CI-02-218R3. PubMed PMID: WOS:000221336700006.

44. Winterbottom R. Search for the gobioid sister group (Actinopterygii: Percomorpha). B Mar Sci. 1993;52(1):395–414. PubMed PMID: WOS:A1993KX23000014.

45. Maugé AL. Diagnoses préliminaires d'Eleotridae des eaux douces de Madagascar. Cybium (Paris). 1984;18(4):98-100.

46. Allen GR. Suborder Kurtoidei. In: Carpenter KE, Niem VH, editors. FAO species identification guide for fishery purposes The living marine resources of the Western Central Pacific Volume 6 Bony fishes part 4 (Labridae to Latimeriidae), estuarine crocodiles, sea turtles, sea snakes and marine mammals. 6. Rome: FAO; 2001. p. 3610.

47. Nelson JS. Some Characters of Trichonotidae, with Emphasis to Those Distinguishing It from Creediidae (Perciformes, Trachinoidei). Jpn J Ichthyol. 1986;33(1):1-6. PubMed PMID: WOS:A1986C756900001.

48. Nelson JS. Trichonotidae. In: Carpenter KE, Niem VH, editors. FAO species identification guide for fishery purposes The living marine resources of the Western Central Pacific Volume 6 Bony fishes part 4 (Labridae to Latimeriidae), estuarine crocodiles, sea turtles, sea snakes and marine mammals. 6. Rome: FAO; 2001. p. 3511-6.

49. Harrison IJ. Specialization of the gobioid palatopterygoquadrate complex and its relevance to gobioid systematics. Journal of Natural History. 1989;23(2):325–53. doi: 10.1080/00222938900770211.

50. Shibukawa K, Iwata A, Viravong S. *Terateleotris*, a new gobioid fish genus from the Laos (Teleostei, Perciformes), with comments on its relationships. Bulletin of the National Science Museum Series A (Zoology). 2001;27(4):229–57. PubMed PMID: BCI:BCI200200292381.

51. Near TJ, Dornburg A, Eytan RI, Keck BP, Smith WL, Kuhn KL, et al. Phylogeny and tempo of diversification in the superradiation of spiny-rayed fishes. Proc Natl Acad Sci U S A. 2013;110(31):12738–43. doi: 10.1073/pnas.1304661110. PubMed PMID: 23858462; PubMed Central PMCID: PMC3732986.

52. Reichenbacher B, Cappetta H. First evidence of an early Miocene marine teleostean fish fauna (otoliths) from La Paillade (Montpellier, France). Palaeovertebrata. 1999;28(1):1–46.

53. Reichenbacher B, Codrea V. Fresh- to brackish water fish faunas from continental Early Oligocene deposits in the Transylvanian Basin (Romania). Bull de l'Institut Roy Sci Nat Belgique. 1999;69:197–207.

54. Reichenbacher B, Weidmann M. Fisch-Otolithen aus der oligo-/miozänen Molasse der West-Schweiz und der Haute-Savoie (Frankreich). Stuttgarter Beitr Naturk. 1992;184:1–83. PubMed PMID: BCI:BCI199395071118.

Author Contributions

Conceptualization: Bettina Reichenbacher, Tomáš Přikryl.

Data curation: Bettina Reichenbacher, Tomáš Přikryl, Alexander F. Cerwenka.

Formal analysis: Bettina Reichenbacher, Tomáš Přikryl, Christoph Gierl, Martin Dohrmann.

Funding acquisition: Bettina Reichenbacher, Tomáš Přikryl.

Investigation: Bettina Reichenbacher, Tomáš Přikryl.

Methodology: Bettina Reichenbacher, Tomáš Přikryl, Alexander F. Cerwenka, Christoph Gierl, Martin Dohrmann.

Resources: Bettina Reichenbacher, Tomáš Přikryl, Alexander F. Cerwenka, Philippe Keith.

Supervision: Bettina Reichenbacher, Tomáš Přikryl.

Visualization: Bettina Reichenbacher, Tomáš Přikryl, Alexander F. Cerwenka.

Writing – original draft: Bettina Reichenbacher, Tomáš Přikryl.

Writing – review & editing: Bettina Reichenbacher, Tomáš Přikryl, Christoph Gierl, Martin Dohrmann, Alexander F. Cerwenka, Philippe Keith.

Figure1

Click here to access/download;Figure;Fig1_LZW.tif

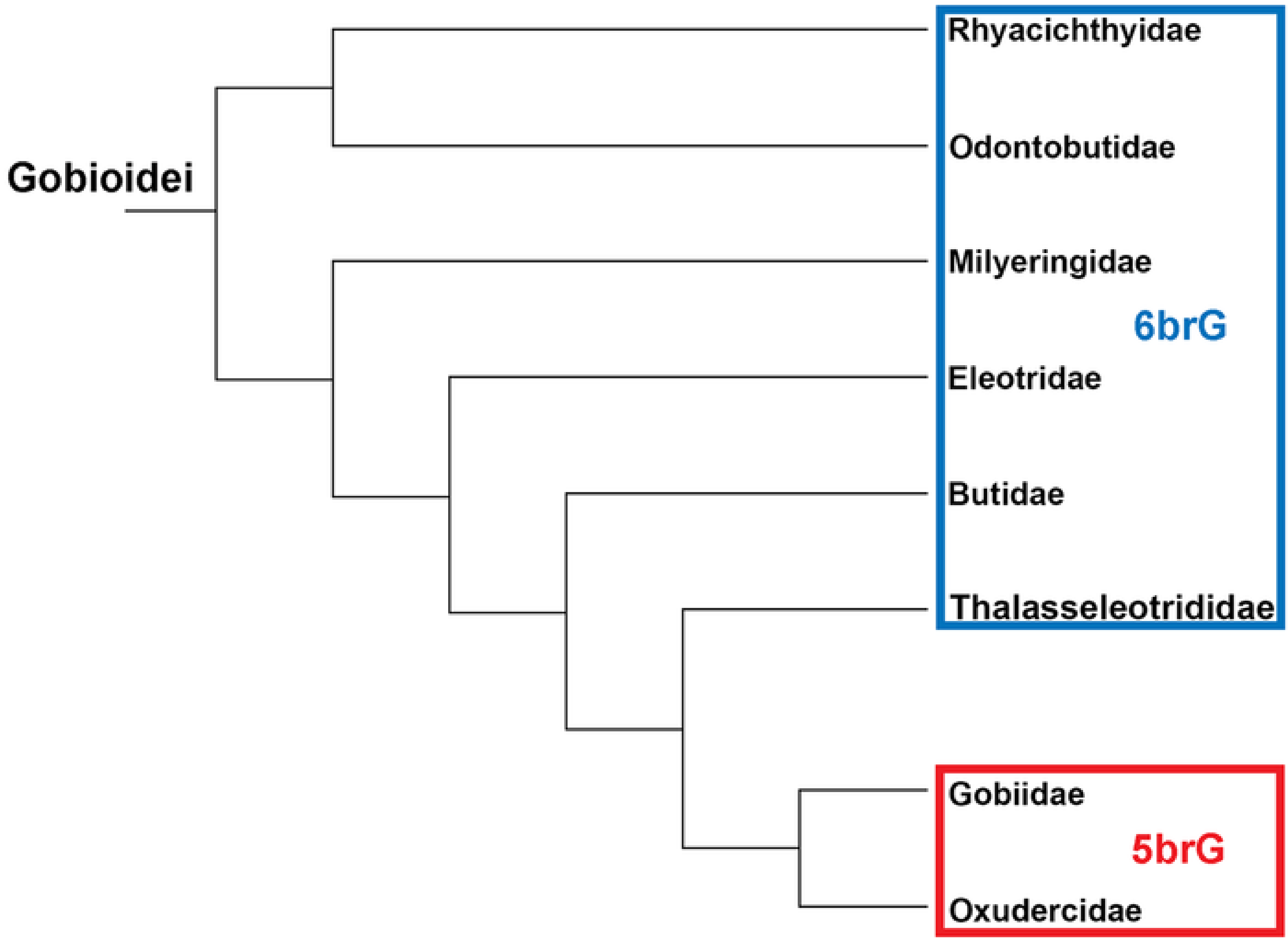

Figure2

Click here to access/download;Figure;Fig2_LZW.tif

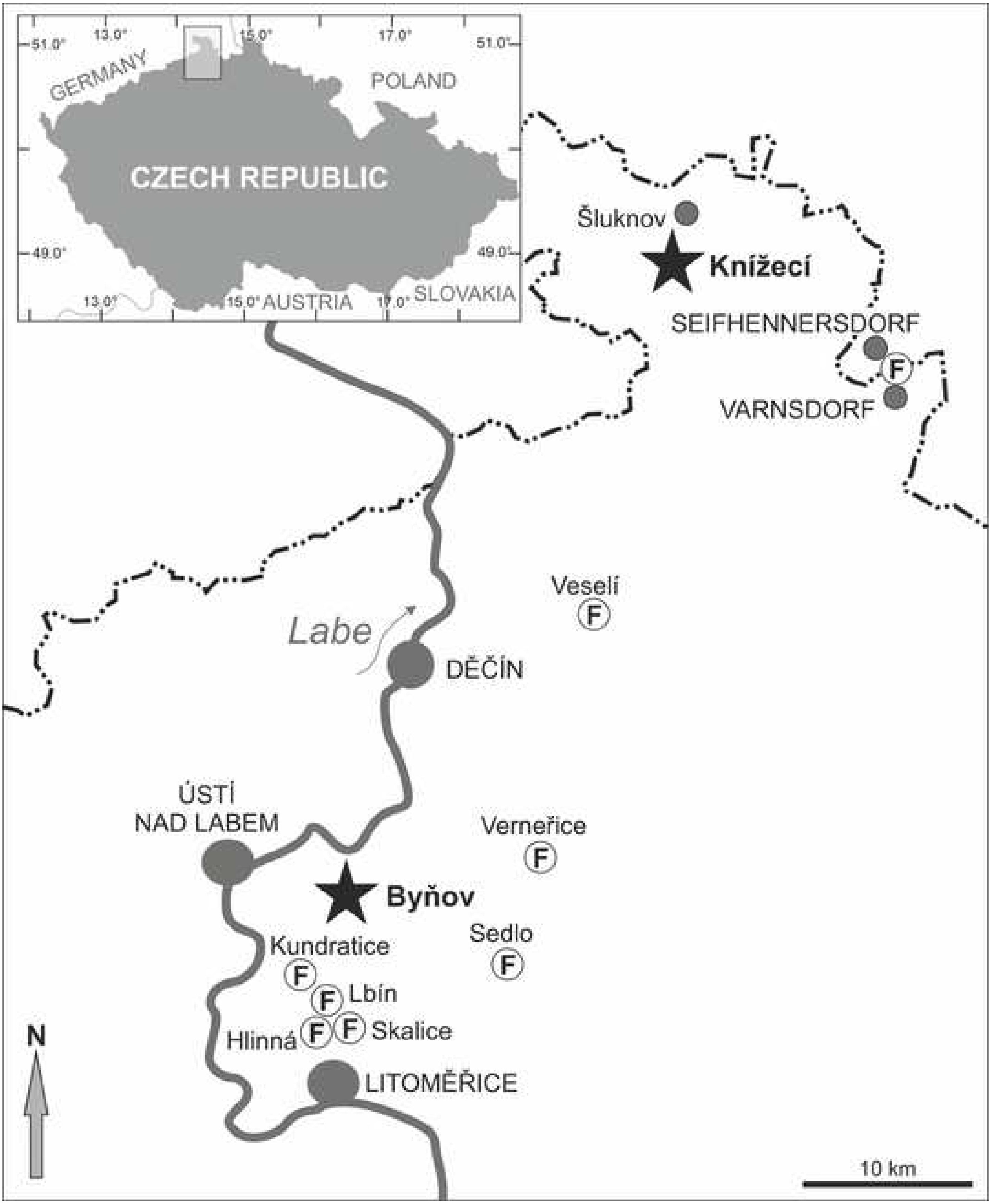

Figure3

Click here to access/download;Figure;Fig3_LZW.tif

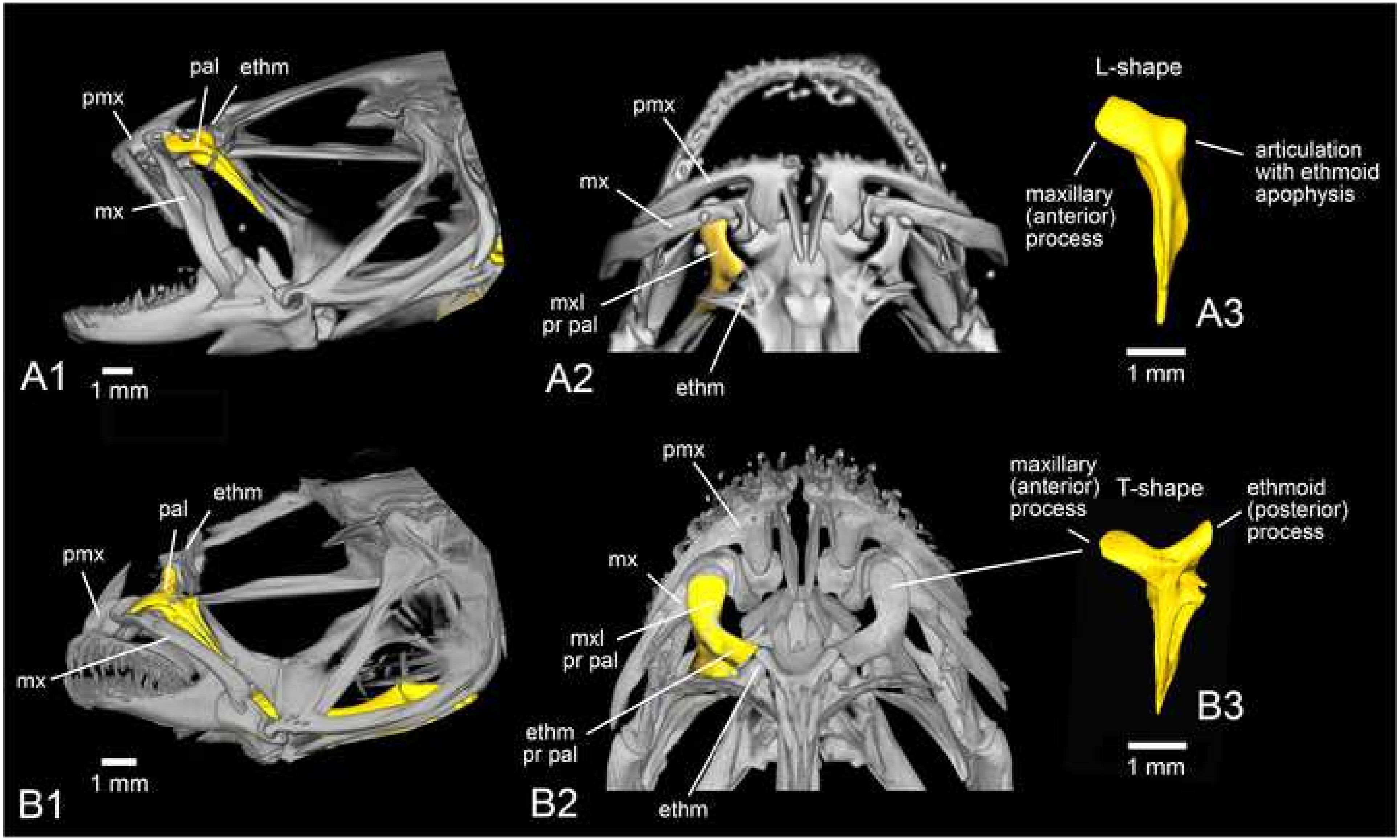

Figure4

Click here to access/download;Figure;Fig4_LZW.tif

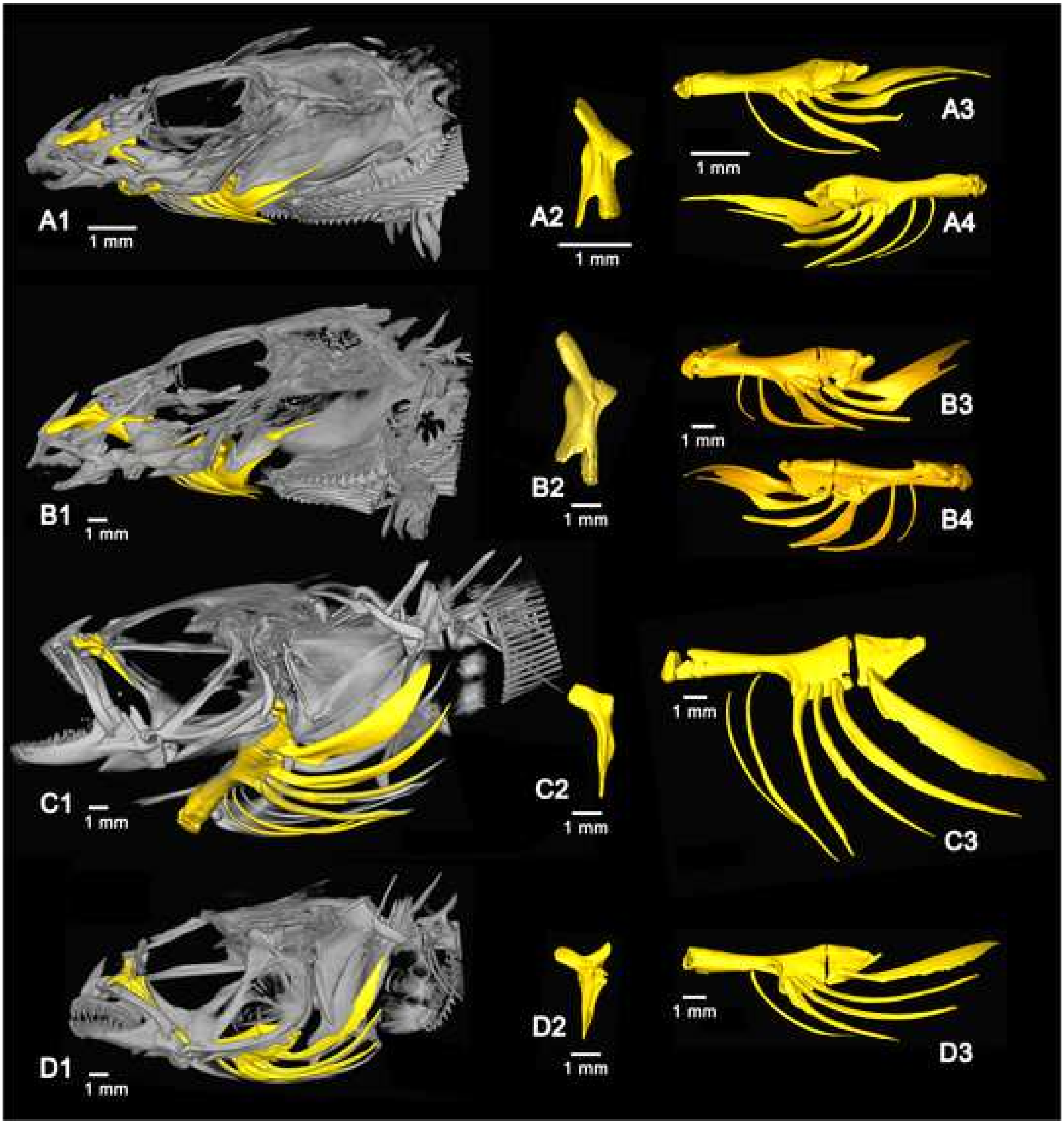

Figure5

Click here to access/download;Figure;Fig5_LZW.tif

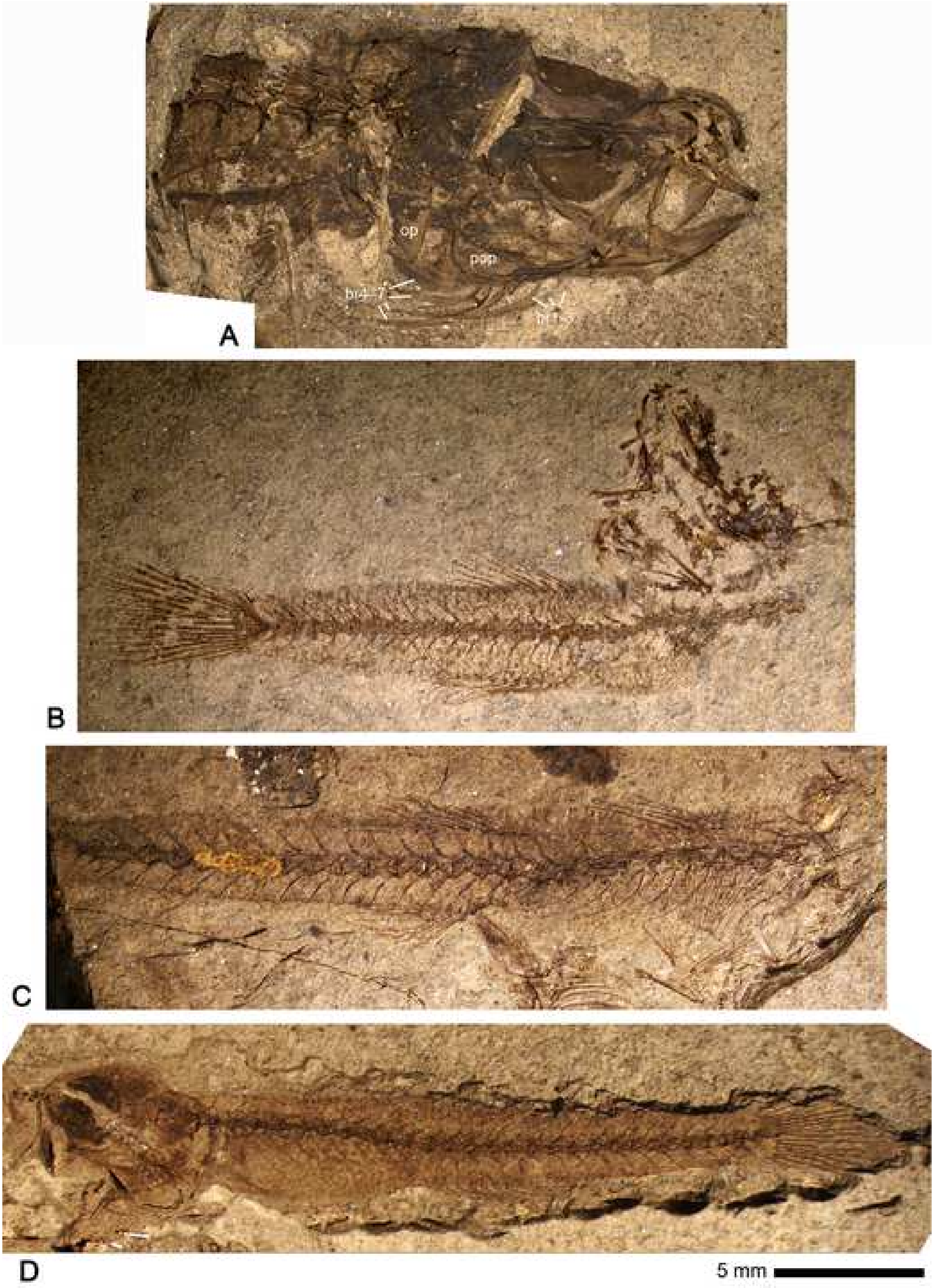

Figure6

Click here to access/download;Figure;Fig6_LZW.tif

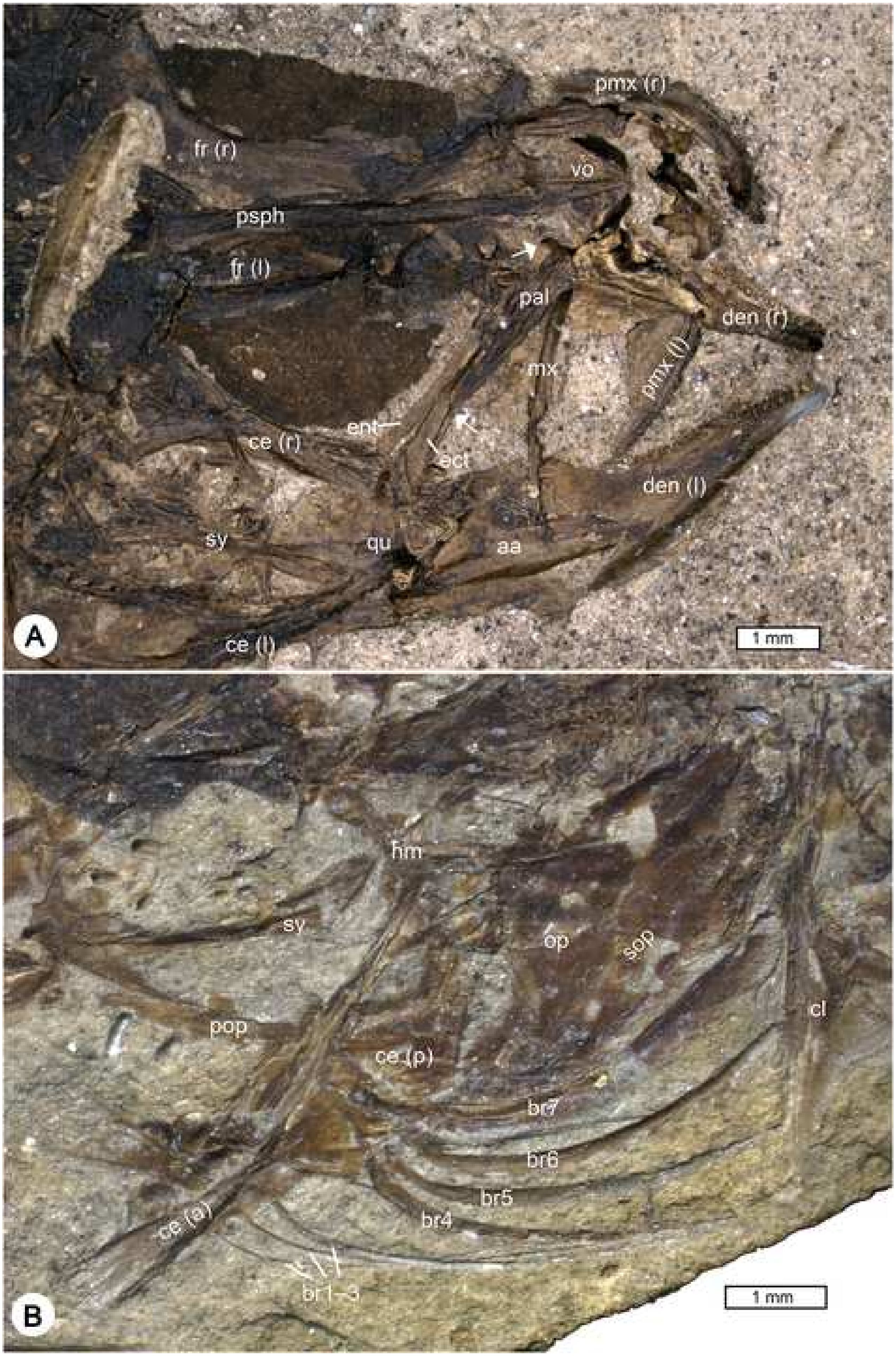

Figure7 Click here to access/download;Figure;Fig7_LZW.tif

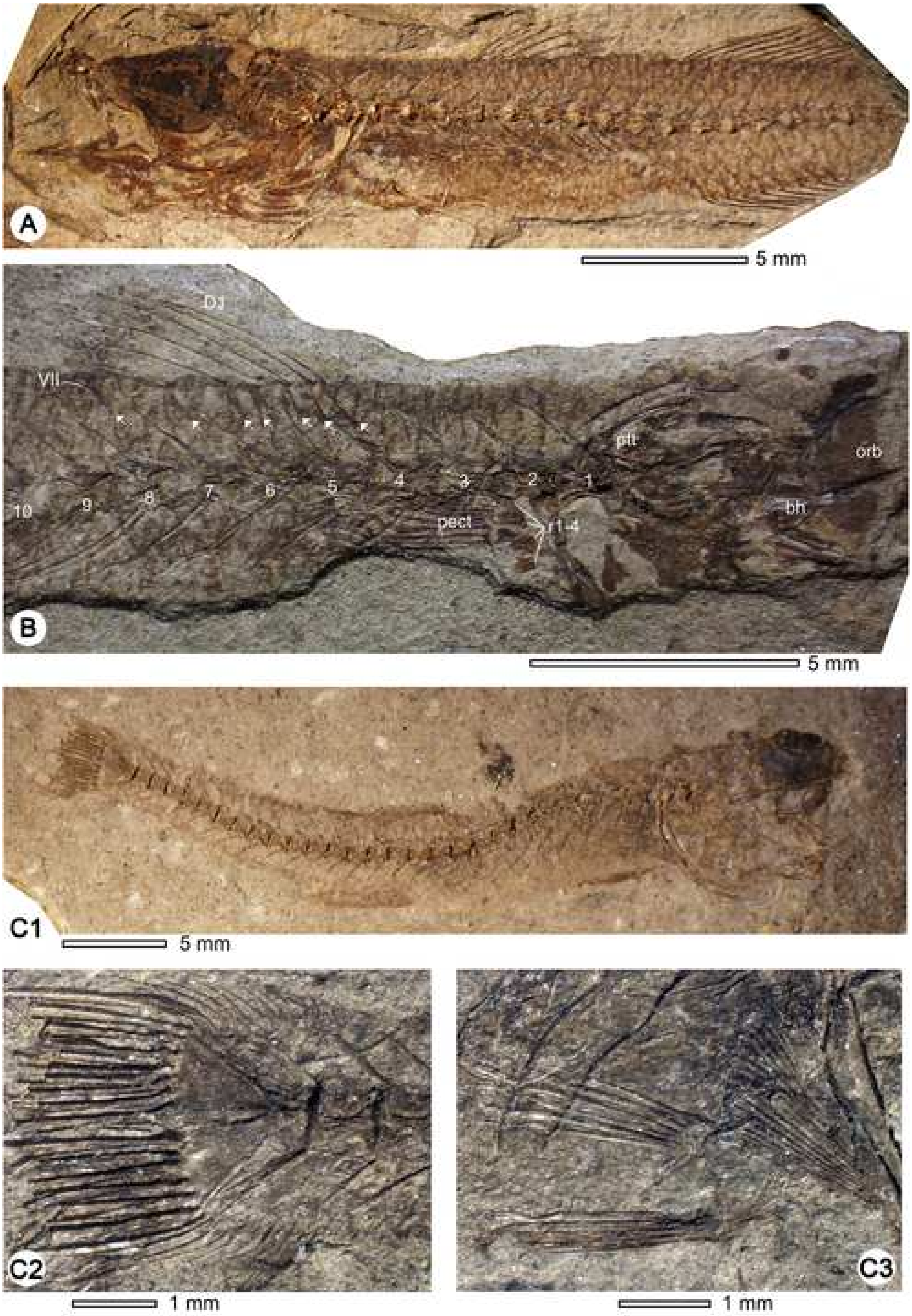

Figure8 Click here to access/download;Figure;Fig8_LZW.tif

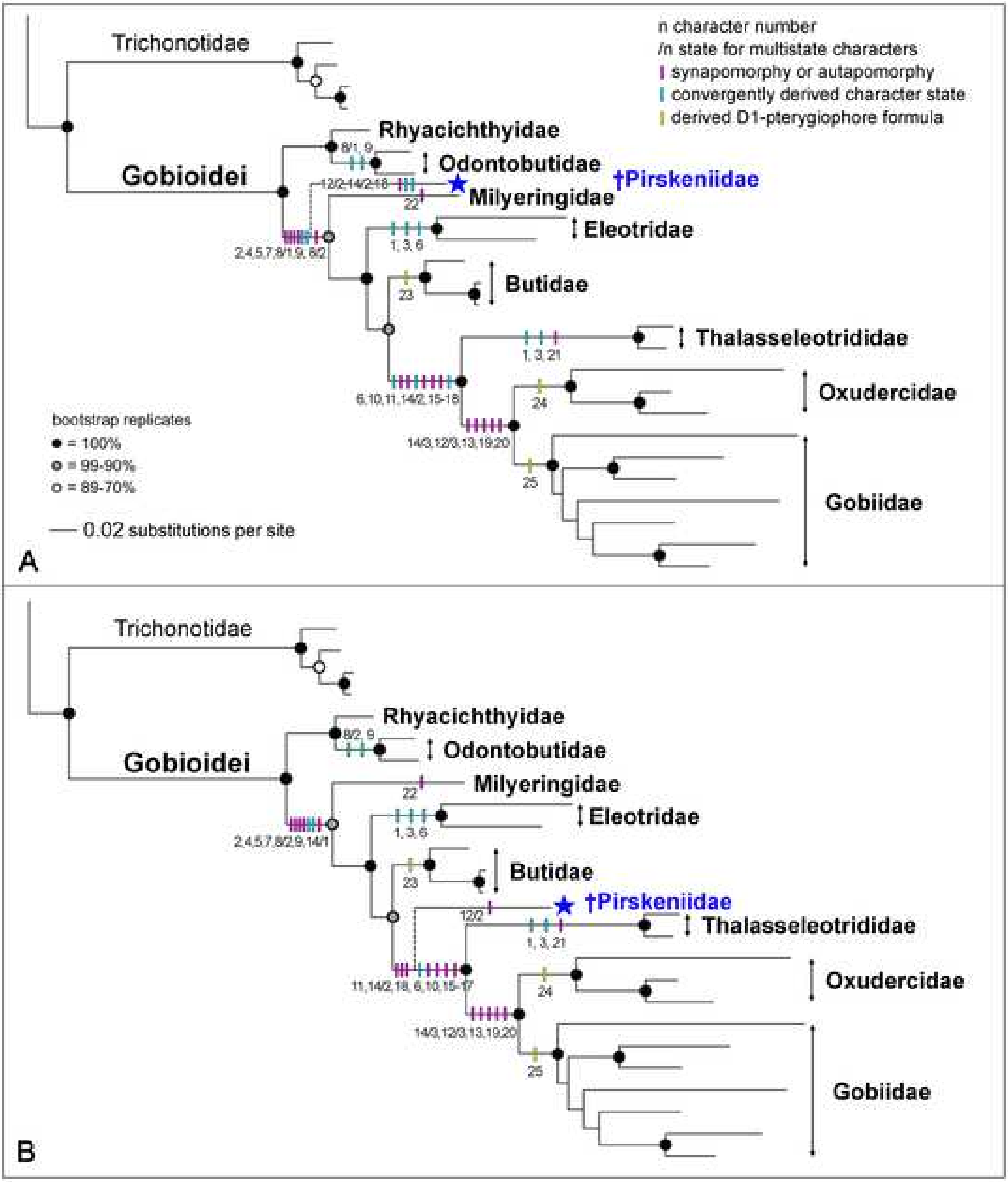

 Click here to access/download;Figure;Fig9_LZW.tif

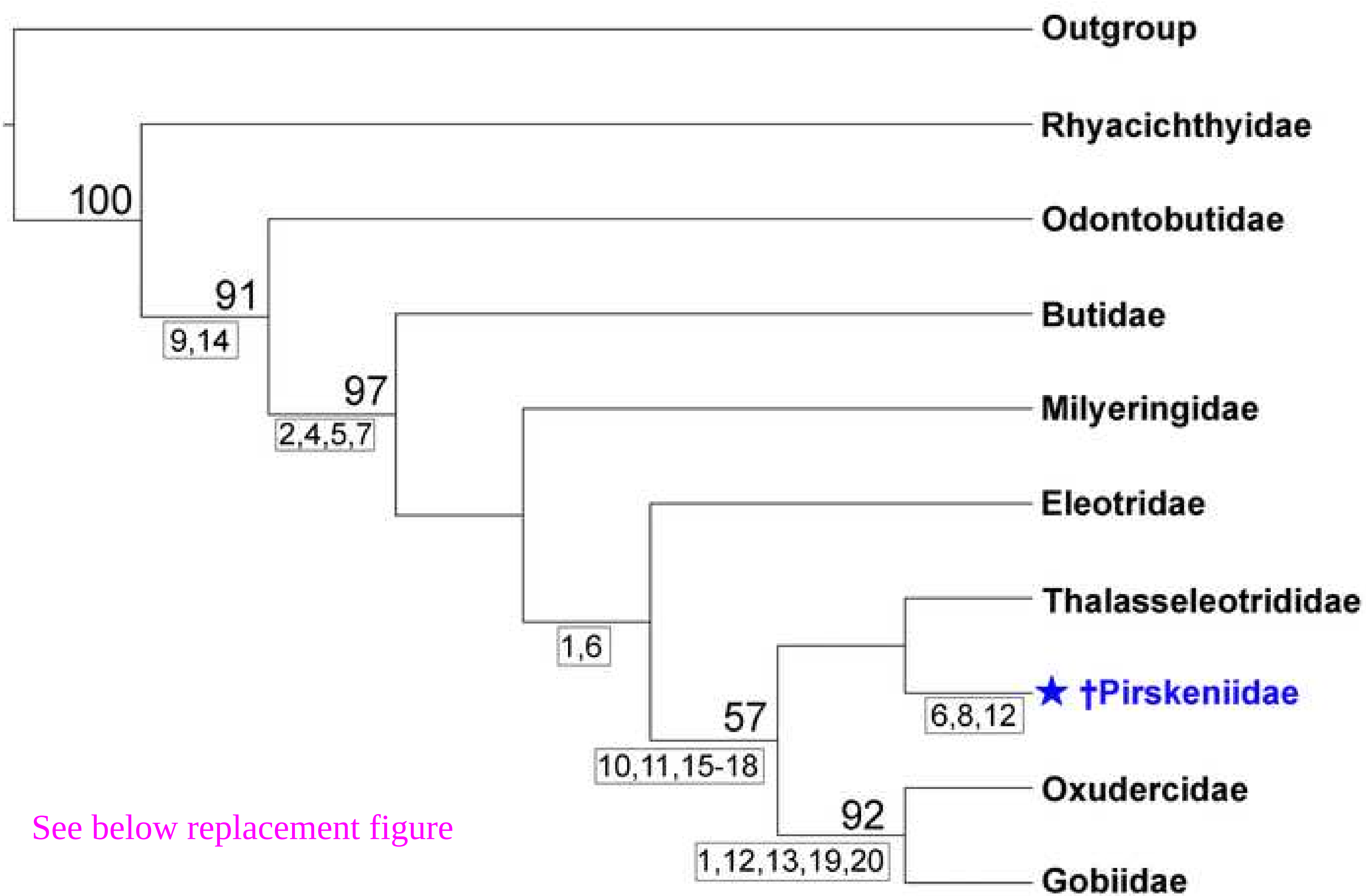

See below replacement figure

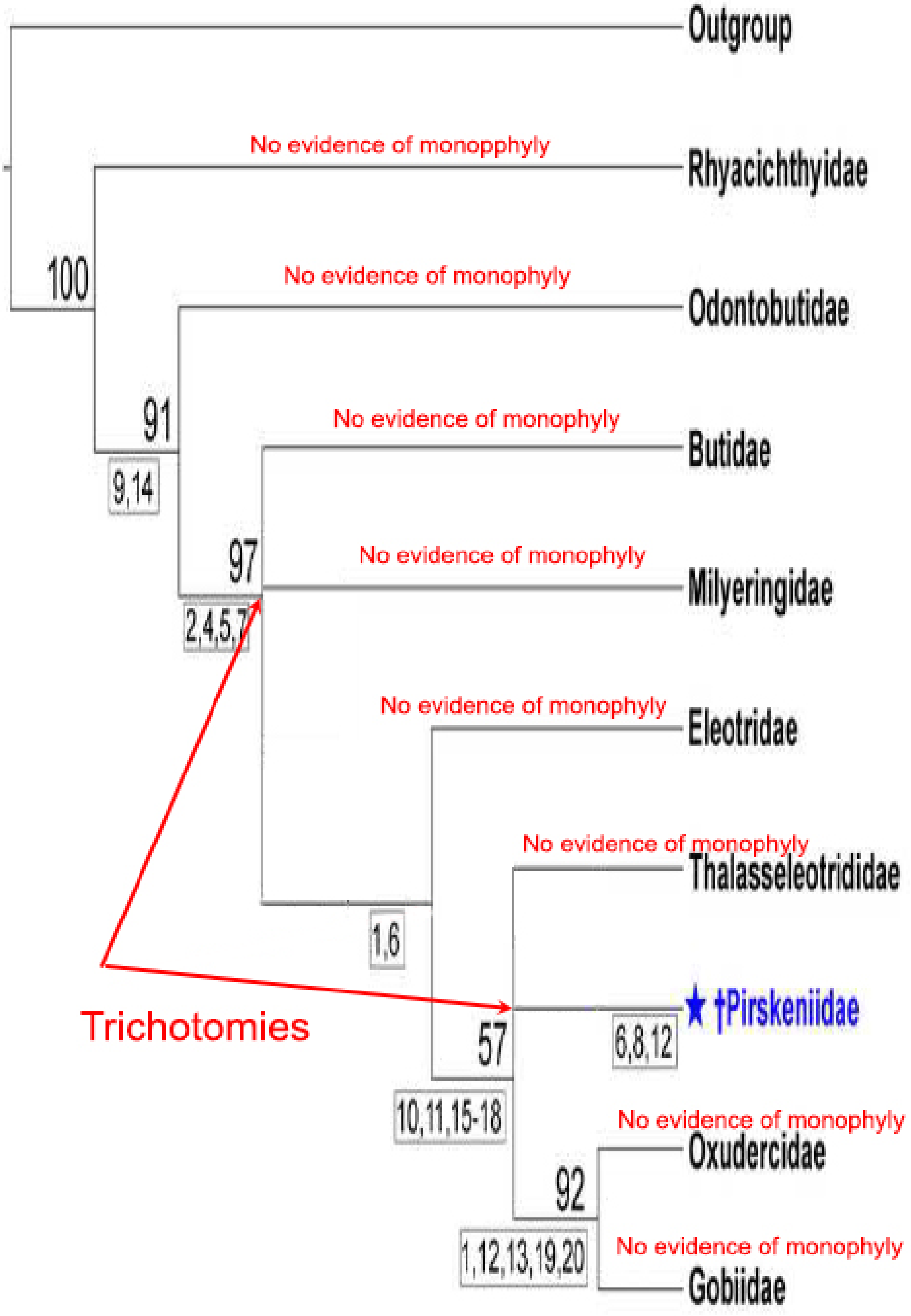

Outgroup
No evidence of monopphyly
Rhyacichthyidae
100
No evidence of monophyly
Odontobutidae
91
9,14
No evidence of monophyly
Butidae
97
No evidence of monophyly
Milyeringidae
2,4,5,7
No evidence of monophyly
Eleotridae
No evidence of monophyly
Thalasseleotrididae
1,6
Trichotomies
★ †Pirskeniidae
6,8,12
57
10,11,15-18
No evidence of monophyly
Oxudercidae
92
1,12,13,19,20
No evidence of monophyly
Gobiidae

Click here to access/download;Figure;Table1.docx

Table 1. Technical details of the micro-CTs of extant gobioids.

	Voltage [kV]	Current [A]	Voxel resolution [µm]	Number of scans and projections per scan
Rhyacichthys guilberti MNHN 2019-0113 (small specimen)	180	125	19.6	3*1600
Rhyacichthys guilberti MNHN 2019-0113	100	140	9.3	2*1440
Eleotris pisonis ZSM 9393, 41704	100	100	4.1	1*1200
Gobius incognitus NMP 6V 146150	110	50	16.1	3*1440

Table 2. Morphological characters for classification of Gobioidei.

Characters that refer to soft tissue or delicate bony structures and thus are unlikely to be preserved in a fossil are indicated in italics. Characters 21–25 refer to autapomorphies.

Description of characters and states	Outgroup	Rhyacichthyidae	Odontobutidae	Milyeringidae	Eleotridae	Butidae	Thalasseleotrididae	Gobiidae	Oxudercidae	†Pirskeniidae
(1) *adductor mandibulae tendon attachment* (Hoese & Gill 1993: char. 1, modified from binary to multistate character)*:* 0 = posterior to or on maxilla head; 1 = on posterodorsolateral margin of maxilla; 2 = to short process extending from maxilla just posterior to maxilla head; 3 = medially halfway down directly at maxilla shaft; 4 = various attachments	0	1	2	2	3	2	3	4	4	?
(2) *anterior extent of procurrent cartilages* (Hoese & Gill 1993: char. 2): 0 = small; 1 = expanded	0	0	0	1	1	1	1	1	1	?
(3) *posterior extent of procurrent caudal cartilages* (Hoese & Gill 1993: char. 3): 0 = relatively short; 1 = elongated										
(4) scapula/radial position (Hoese & Gill 1993: char. 4): 0 = radial separate from cleithrum, large ossified scapula; 1 = radial adjoins cleithrum, scapula unossified or reduced	0	0	0	1	1	1	1	1	1	1
(5) middle radial of first pterygiophore of second dorsal fin (Hoese & Gill 1993: char. 5): 0 = present; 1 = fused with proximal radial (or lost)	0	0	0	1	1	1	1	1	1	?
(6) bony preopercle canal support (Hoese & Gill 1993: char. 6): 0 = usually extends full length of preopercle; 1 = confined to vertical portion of preopercle or absent	0	0	0	0	1	0	1	1	1	0
(7) ctenoid scales with transforming ctenii (Hoese & Gill (1993: char. 7): 0 = present; 1 = absent	0	0	0	1	1	1	1	1	1	1
(8) penultimate branchiostegal ray position (Hoese & Gill 1993: char. 8; this study, modified from binary to multistate character): 0 = at posterior ceratohyal; 1 = at anterior ceratohyal just before gap to posterior ceratohyal; 2 = at anterior ceratohyal anterior to gap to posterior ceratohyal	0	0	1	2	2	2	2	2	2	1
(9) lateral line (Hoese & Gill 1993: char. 9): 0 = present; 1 = absent	0	0	1	1	1	1	1	1	1	1
(10) ventral shelf on urohyal (Hoese & Gill 1993: char. 12; Gill & Mooi 2012): 0 = present; 1 = absent	0	0	0	0	0	0	1	1	1	?
(11) interneural gap between last pterygiophore of first dorsal fin and first pterygiophore of second dorsal fin (Hoese & Gill 1993: char. 13): 0 = absent; 1 = present	0	0	0	0	0	0	1	1	1	1
(12) serial number of expanded last branchiostegal ray (Hoese & Gill 1993: char. 15; Gill & Mooi 2012; this study and modified from binary to multistate character): 0 = absent; 1 = sixth ray; 2 = seventh ray; 3 = fifth ray	0	1	1	1	1	1	1	3	3	2
(13) Pelvic fins (Akihito 2000; Hoese & Gill 1993: char. 16): 0 = separated; 1 = united	0	0	0	0	0	0	0	1	1	0
(14) palatine shape (Regan 1911; Hoese 1984; this study): 0 = inconstant; 1 = L-shaped; 2 = not exactly L-shaped; 3 = T-shaped	0	0	1	1	1	1	2	3	3	2
(15) *Interhyal with cup-shaped lateral structure for articulation with preopercle* (Gill & Mooi 2012): 0 = not developed; 1 = present	0	0	0	0	0	0	1	1	1	?
(16) *laterally-directed posterior process on the posterior ceratohyal supporting the interhyal* (Gill & Mooi 2012): 0 = not developed; 1 = present	0	0	0	0	0	0	1	1	1	?
(17) *Pharyngobranchial 4 absent, epibranchial 4 directly articulating with pharyngobranchial 3* (Gill & Mooi 2012): 0 = not developed; 1 = present	0	0	0	0	0	0	1	1	1	?
(18) dorsal postcleithrum (Gill & Mooi 2012): 0 = present, 1 = absent	0	0	0	0	0	0	1	1	1	1
(19) *expanded and medially-placed ventral process on ceratobranchial 5* (Gill & Mooi 2012): 0 = not developed; 1 = present	0	0	0	0	0	0	0	1	1	?
(20) *dorsal hemitrich of pelvic-fin rays with complex proximal head* (Gill & Mooi 2012): 0 = not developed; 1 = present	0	0	0	0	0	0	0	1	1	?
(21) *Membrane connecting hyoid arch to ceratobranchial 1 broad and extending most of length of ceratobranchial 1* (Gill & Mooi 2012): 0 = not developed; 1 = present	0	0	0	0	0	0	1	0	0	?
(22) eyes (Whitley 1945): 0 = present, 1 = absent	0	0	0	1	0	0	0	0	0	0
(23) D1 pterygiophore formula mostly 3-2211 (Gierl et al. 2013): 0 = not developed; 1 = present	0	0	0	0	0	1	0	0	0	0
(24) D1 pterygiophore formula mostly 3-22110 (Birdsong et al. 1988; Reichenbacher et al. 2018): 0 = not developed; 1 = present.	0	0	0	0	0	0	0	1	0	0
(25) D1 pterygiophore formula mostly 3-12..00 (Birdsong et al. 1988; McKay & Miller 1997): 0 = not developed; 1 = present.	0	0	0	0	0	0	0	0	1	0

Table 3. Morphometric data of †*Pirskenius diatomaceus*.

Specimens are arranged according to their standard length (SL). Values depict measurements in mm, values in brackets refer to % SL, only in case of eye diameter (ED) to % of head length (HL). * indicates that SL was calculated based on measurement of body length (BL) or HL or caudal peduncle length (CPL) by assuming a proportion of BL = 76% of SL, HL = 24% of SL, and CPL = 32% SL, respectively, based on measurements of complete specimens. For abbreviations see Material and methods.

	PC 2777	PC 2813	Pv 11672	PC 2787	PC 2794/2795	PC 2799	Pv 11671	PC 2779	PC 2772	PC 2791	Pv 11669
**SL**	23.2	23.4*	24.9	26.2*	30.5	30.5	34.1*	35.3*	35.8*	36.6*	55.4*
**HL**	5.4 (23.3)		6.0 (24.0)	6.3	7.2 (23.6)	7.5 (24.6)			8.6	(8.8)	13.3
**ED**	2.0 (37.0)			1.9 (30.1)	2.7 (37.5)	2.4 (32.0)			2.9 (33.7)	2.4 (27.3)	3.6 (27.0)
**BL**	17.8 (76.7)		18.9 (75.9)		23.3 (76.4)	23 (75.4)	25.9	26.8		27.8	
**BH**	2.2 (9.4)		c. 3.2 (12.8)		3.5 (11.5)	3.9 (12.8)		3.5 (9.9)	4.7 (13.1)	c. 4.5 (12.3)	
**SN/D1**	-		9.1 (36.5)			11.6 (38.0)			13.2 (36.9)		
**SN/D2**	12.4 (53.4)		13.7 (55.0)		c. 16 (c. 52.4)	16.8 (55.1)			19.8 (55.3)		
**SN/A**	13.6 (58.6)		14.2 (57.0)		c. 17.1 (56.1)	17.4 (57.0)			20.8 (58.1)		
**LPect**									c. 5.0 (14.0)		
**LPelv**									4.5 (12.6)		
**D2 base**		3.6 (15.4)	3.5 (14.0)				4.6 (13.5)	4.6 (13.0)	4.5 (12.6)	c. 4.9 (13.4)	
**A base**		3.5 (14.9)	3.3 (13.2)		3.1 (10.2)	3.3 (10.8)	4.0 (11.7)	4.2 (11.9)	4.2 (11.7)	3.7 (10.1)	
**CPL**	5.7? (24.6)	7.5	7.2 (28.9)		c. 9.8 (32.1)	9.9 (32.4)	11.5 (33.7)	11.8 (33.4)		11.8 (32.2)	
**CPH**	1.8 (7.7)	2.7 (11.5)	2.9 (11.6)		3.1 (10.2)	2.9 (9.5)	c. 3.2 (9.4)	3.8 (10.8)		3.3 (9.0)	
**CPL/CPH**	3.1	2.75	2.5		3.2	3.4	3.6	3.1		3.6	
**D2C**		7.3 (31.2)	6.6 (26.5)		9.7 (31.8)		10.0 (29.3)	10.2 (28.9)		9.0 (24.5)	

 Click here to access/download;Figure;Table4.docx

Table 4. Meristic data of †*Pirskenius diatomaceus*.

Specimens are arranged according to their collection numbers. For abbreviations see Material and methods.

	PC 2771	PC 2772/ 2773	PC 2774	PC 2779	PC 2781	PC 2788	PC 2791	PC 2794/ 2795	PC 2796	PC 2799	PC 2804	PC 2809	Pv11671	Pv11672
**abd vert**		11		11		11	11	11	11	11			11	11
**caud vert**				17		17	18	17	17	17			16	16
**D1**	VI	VII	VII	VII		VI		VII			VI		VII	VII
**D2**		I9				I10		I10			I10		I9	I9
**D1 pt-form. 3(122110)**		+	+	+										
**D1 pt-form. 3(12210)**						+								
**A**		I9				I10				I9			I9	I10
**AP**		2					3							2
**Pect**											12-13		13-14	
**Pelv**			I5								I5		I5	
**C (dorsal/ventral)**					7/6	7/6	7/6	7/6	7/7			7/6		7/6
**dorsal prC**					>7	11	12	>7	>7			14	11	11
**ventral prC**					9	11	8	11	c. 8			10		9
**cycloid scales on hypurals**					+	+	+	+				+	+	

Table 5. Comparison of morphometric data (in % of SL) and meristic counts between the two species of †*Pirskenius*.

Ranges and counts for †*P. diatomaceus* refer to the values provided in Tables 3 and 4, data for †*P. radoni* originates from Přikryl [16] and this study. For abbreviations see Material and methods.

	†P. diatomaceus	***†P. radoni***
**SL**	23.2–55.4	17.8 | 34.0
**HL**	23.3–24.6	28.7 | 32.4
**BH**	9.4–13.1	- | 19.4
**SN/D1**	36.5–38.0	45.5 | 39.4
**SN/D2**	52.4–55.3	- | 59.4
**SN/A**	57.0–58.1	62.4 | 59.7
**CPL**	28.9–33.7	- | 25.3
**CPH**	7.7–11.6	7.9 | 9.1
**branchiostegal rays**	7	7
**abd vert**	11	12
**caud vert**	16–17(18)	16
**D1**	VI–VII	VII
**D2**	I9–10	I8
**D1 pt-form. 3(122110)**	+	
**D1 pt-form. 3(12210)**	+	
**D1 pt-form. (4-32110)**		+
**A**	I9–10	I9
**AP**	2–3	4
**Pect**	12–14	>12
**Pelv**	I5	I5
**prC (dorsal/ventral)**	7/6 (7/7)	8/6
