# Supplementary material for: Freshwater gobies 30 million years ago: New insights into character evolution and phylogenetic relationships of †Pirskeniidae (Gobioidei, Teleostei)
Source: PLoS One. 2020 Aug 24;15(8):e0237366. doi: 10.1371/journal.pone.0237366 (PMC7446829; doi:10.1371/journal.pone.0237366)
Supplement: Attachment — Submitted filename: Response to PONE Reviewers comments.docx [file pone.0237366.s006.docx]

PONE-D-20-14246
Freshwater gobies 30 million years ago: new insights into character evolution and phylogenetic relationships of †Pirskeniidae (Gobioidei, Teleostei)
PLOS ONE

Dear Dr. Reichenbacher,

Thank you for submitting your manuscript to PLOS ONE. After careful consideration, we feel that it has merit but does not fully meet PLOS ONE’s publication criteria as it currently stands. Therefore, we invite you to submit a revised version of the manuscript that addresses the points raised during the review process.


Please submit your revised manuscript by Jul 23 2020 11:59PM. If you will need more time than this to complete your revisions, please reply to this message or contact the journal office at plosone@plos.org. When you're ready to submit your revision, log on to https://www.editorialmanager.com/pone/ and select the 'Submissions Needing Revision' folder to locate your manuscript file.

Please include the following items when submitting your revised manuscript:

- A rebuttal letter that responds to each point raised by the academic editor and reviewer(s). You should upload this letter as a separate file labeled 'Response to Reviewers'.
- A marked-up copy of your manuscript that highlights changes made to the original version. You should upload this as a separate file labeled 'Revised Manuscript with Track Changes'.
- An unmarked version of your revised paper without tracked changes. You should upload this as a separate file labeled 'Manuscript'.

If you would like to make changes to your financial disclosure, please include your updated statement in your cover letter. Guidelines for resubmitting your figure files are available below the reviewer comments at the end of this letter.

If applicable, we recommend that you deposit your laboratory protocols in protocols.io to enhance the reproducibility of your results. Protocols.io assigns your protocol its own identifier (DOI) so that it can be cited independently in the future. For instructions see: <http://journals.plos.org/plosone/s/submission-guidelines#loc-laboratory-protocols>

We look forward to receiving your revised manuscript.

Kind regards,

Giorgio Carnevale, Ph.D
Academic Editor
PLOS ONE


Additional Editor Comments:

Both the reviewers considered the manuscript an interesting contribution but also evidenced a number of issues that must be necessarily addressed.

Journal requirements:

When submitting your revision, we need you to address these additional requirements.

1. Please ensure that your manuscript meets PLOS ONE's style requirements, including those for file naming. The PLOS ONE style templates can be found at

https://journals.plos.org/plosone/s/file?id=wjVg/PLOSOne_formatting_sample_main_body.pdf and PLOSOne_formatting_sample_main_body.pdf Done

https://journals.plos.org/plosone/s/file?id=ba62/PLOSOne_formatting_sample_title_authors_affiliations.pdf
Done

2. Please include your tables as part of your main manuscript and remove the individual files. Please note that supplementary tables should be uploaded as separate "supporting information" files. Done

3. Thank you for stating the following in the Acknowledgments Section of your manuscript:

'We acknowledge funding for this project from the Deutsche Forschungsgemeinschaft to B.R. (RE-1113/20). T.P.’s research was institutionally supported by the Czech Academy of the Sciences, Institute of Geology (RVO67985831).'

We note that you have provided funding information that is not currently declared in your Funding Statement. However, funding information should not appear in the Acknowledgments section or other areas of your manuscript. We will only publish funding information present in the Funding Statement section of the online submission form.

Please remove any funding-related text from the manuscript and let us know how you would like to update your Funding Statement. Currently, your Funding Statement reads as follows:

'he funders had no role in study design, data collection and analysis, decision to publish, or preparation of the manuscript.'

Please include your amended statements within your cover letter; we will change the online submission form on your behalf.

 Done

4. We note that Figure 2 in your submission contains map images which may be copyrighted.

All PLOS content is published under the Creative Commons Attribution License (CC BY 4.0), which means that the manuscript, images, and Supporting Information files will be freely available online, and any third party is permitted to access, download, copy, distribute, and use these materials in any way, even commercially, with proper attribution. For these reasons, we cannot publish previously copyrighted maps or satellite images created using proprietary data, such as Google software (Google Maps, Street View, and Earth). For more information, see our copyright guidelines: http://journals.plos.org/plosone/s/licenses-and-copyright.

We require you to either (a) present written permission from the copyright holder to publish this figure specifically under the CC BY 4.0 license, or (b) remove the figure from your submission:

a. You may seek permission from the original copyright holder of Figure 2 to publish the content specifically under the CC BY 4.0 license.

We recommend that you contact the original copyright holder with the Content Permission Form ([http://journals.plos.org/plosone/s/file?id=7c09/content-permission-form.pdf](about:blank)) and the following text:

“I request permission for the open-access journal PLOS ONE to publish XXX under the Creative Commons Attribution License (CCAL) CC BY 4.0 ([http://creativecommons.org/licenses/by/4.0/](about:blank)). Please be aware that this license allows unrestricted use and distribution, even commercially, by third parties. Please reply and provide explicit written permission to publish XXX under a CC BY license and complete the attached form.”

Please upload the completed Content Permission Form or other proof of granted permissions as an "Other" file with your submission.

In the figure caption of the copyrighted figure, please include the following text: “Reprinted from [ref] under a CC BY license, with permission from [name of publisher], original copyright [original copyright year].” Done

b. If you are unable to obtain permission from the original copyright holder to publish this figure under the CC BY 4.0 license or if the copyright holder’s requirements are incompatible with the CC BY 4.0 license, please either i) remove the figure or ii) supply a replacement figure that complies with the CC BY 4.0 license. Please check copyright information on all replacement figures and update the figure caption with source information. If applicable, please specify in the figure caption text when a figure is similar but not identical to the original image and is therefore for illustrative purposes only.

The following resources for replacing copyrighted map figures may be helpful:

USGS National Map Viewer (public domain): [http://viewer.nationalmap.gov/viewer/](about:blank)

The Gateway to Astronaut Photography of Earth (public domain): [http://eol.jsc.nasa.gov/sseop/clickmap/](about:blank)

Maps at the CIA (public domain): [https://www.cia.gov/library/publications/the-world-factbook/index.html](about:blank) and [https://www.cia.gov/library/publications/cia-maps-publications/index.html](about:blank)

NASA Earth Observatory (public domain): [http://earthobservatory.nasa.gov/](about:blank)

Landsat: http://landsat.visibleearth.nasa.gov/

USGS EROS (Earth Resources Observatory and Science (EROS) Center) (public domain): [http://eros.usgs.gov/#](about:blank)

Natural Earth (public domain): [http://www.naturalearthdata.com/](about:blank)

5. Your ethics statement must appear in the Methods section of your manuscript. If your ethics statement is written in any section besides the Methods, please move it to the Methods section and delete it from any other section. Please also ensure that your ethics statement is included in your manuscript, as the ethics section of your online submission will not be published alongside your manuscript.

[Note: HTML markup is below. Please do not edit.]

Reviewers' comments:

Reviewer's Responses to Questions

**Comments to the Author**

1. Is the manuscript technically sound, and do the data support the conclusions?

The manuscript must describe a technically sound piece of scientific research with data that supports the conclusions. Experiments must have been conducted rigorously, with appropriate controls, replication, and sample sizes. The conclusions must be drawn appropriately based on the data presented.

Reviewer #1: Partly

Reviewer #2: Partly

2. Has the statistical analysis been performed appropriately and rigorously?

Reviewer #1: No

Reviewer #2: N/A

3. Have the authors made all data underlying the findings in their manuscript fully available?

The [PLOS Data policy](http://www.plosone.org/static/policies.action#sharing) requires authors to make all data underlying the findings described in their manuscript fully available without restriction, with rare exception (please refer to the Data Availability Statement in the manuscript PDF file). The data should be provided as part of the manuscript or its supporting information, or deposited to a public repository. For example, in addition to summary statistics, the data points behind means, medians and variance measures should be available. If there are restrictions on publicly sharing data—e.g. participant privacy or use of data from a third party—those must be specified.

Reviewer #1: No

Reviewer #2: No

4. Is the manuscript presented in an intelligible fashion and written in standard English?

PLOS ONE does not copyedit accepted manuscripts, so the language in submitted articles must be clear, correct, and unambiguous. Any typographical or grammatical errors should be corrected at revision, so please note any specific errors here.

Reviewer #1: Yes

Reviewer #2: Yes

5. Review Comments to the Author

Please use the space provided to explain your answers to the questions above. You may also include additional comments for the author, including concerns about dual publication, research ethics, or publication ethics. (Please upload your review as an attachment if it exceeds 20,000 characters)

**Reviewer #1:** Authors have done a comprehensive job redescribing the species of Pirskenius and it is good to see an attempt to relate the fossils to existing taxa.

It is not clear why authors used Rhyacichthys. Protogobius or an Odontobutid head scan might have been more informative of primitive conditions. Rhyacichthys is a highly specialised gobioid. Protogobius has different palatine form Rhyacichthys. Some data for Protogobius is in Shibukawa et al.
We used *Rhyacichthys* primarily to demonstrate that exceptions in the 'usual' number of branchiostegal rays can occur, and that such exceptions do not have any phylogenetic meaning. In the revised version, we present a separate Figure for the head scans of Rhyacichthys to explain this better (Fig. 4). As recommended, we added new head scans for *Protogobius* and *Perccottus* (Fig. 5).

With such a large group, authors have selected on 3 taxa to compare the fossil genus, which effectively rules out other groups and makes the character states, which are complex, look overly simple.
We used literature data for further comparison, but we should have mentioned this explicitly. Now we have added at the end of the Introduction the sentence "For further comparisons of †*Pirskenius* and phylogenetic analysis of †Pirskeniidae we used morphological data from previous works [4, 9, 20-29]."

Figure legends are scattered throughout the text, which is confusing.
Figure legends have to be prepared like this according to the Author instructions of PLOS One. We have added a separate file "Figure legends" to facilitate the review of our manuscript.

Figures 5 & 6 are hard to interpret. A drawing showing main points in text would be helpful.
Figure 5 (now Fig. 6) aims to show the new specimens of †*Pirskenius* and their preservation. We would prefer to keep this figure as it is. But we have prepared complementary drawings for Figure 6a (now Fig. 7a, b) and 6b (now Fig. 8a, b).

Table 2. Pterygiophore formula broken into several characters. Primitive appear to be variable and specialisation fixed “mostly” to one state. Essentially a multistate character is being broken into several characters. Also, pterygiophore for Oxudercidae appears incorrect and should be 3-12210 – “mostly”, but see below. Character state for Eleotridae not given, variable but often 3-1221, but in some cases variable within a genus (Allomogurnda and Mogurnda). Appears selective for data analysis. One might argue that the 3(1221) of eleotrids gave rise to oxudercines 3(12210) and that butid 3(2211) gave rise to gobiids 3(22110). In other words different interpretations or codings of character states could give very different results.
Also, it is not clear what usually means in terms of number of species and genera. Also some odontobutids have the same pattern as Pirskenius with lots of variation.
Yes, the variability of the pterygiophore formula makes this character very difficult for a phylogenetic analysis. We have discarded now the pterygiophore formula from Table 2 and the phylogenetic analyes.

Character 1. Adductor mandibular tendon –Hoese & Larson reported the condition in Thlasseleotris as the same as butines, but Hoese & Gill reported the condition as like eleotrids because of the lack of the process on the ~~pre~~maxilla; they did note the tendon in front of the ligament as in butines, although to a lesser degree. Also unfortunately figure 1a & b are reversed in their paper. Reexamination of Thalsseleotris and Grahamichthys indicates a condition more like butines or intermediate, although a subjective calling. In effect it probably should be a character state between 2 and 3, with no process, but tendon above middle of premaxilla.
Thank you for the explanations. We changed the character state for Thalasseleotrididae to "2" (as in Butidae) according to your comments and following the description given in Hoese & Larson (1987). We tested also if the phylogenetic tree changes when we code this character for Thalasseleotridae as additional state (intermediate between 2 and 3), but the tree remains the same.

Character 3 – no data presented. Hoese & Gill mentioned 3 states – not extending over epural, extending over anterior part of epural (in Thalasseleotris and Tateurndina), covering full epural (this state in eleotrids and some gobiids)
'No data' was a mistake in the table, it probably happened during the final editing, we apologize for that. We included the three states according to the detailed description provided in Hoese & Gill (1993).

Character 6 – butid Kribia lacks canals and supports
We have added this to Table 2 and the character matrix

Character 9 – Lateral line present in Terateleotris, considered to be an odontobutid.
We have added this to Table 2 and in the character matrix. The tree remains the same.

Character 11. Interneural gap is present in Rhyacichthyids, some odontobutids, some butids (some Bostrychus) and some eleotrids, although unclear if homologous with gobiid, oxudercid, thalasseleotridid condition.
We have included now the occasional presence of the interneural gap in the Eleotridae and Butidae according to this suggestion.

Character 14. Highly subjective and over simplified. Considerable variation is known to exist in the palatine shape (See Hoese 1984, Harrison, 1989). Gosline also 1958 found variation. Akihito 1969 also showed butine with T-shaped palatine. Palatine is L-shaped in Protogobius, but differently-shaped in Rhyacichthys. Not clear what is meant by inconstant in outgroup.
We have improved the description based on previous works, please see revised Table 2.

Character 18 Dorsal postcleithrum absent in some eleotrids.
We have included now the occasional absence of the dorsal postcleithrum in the Eleotridae, please see revised Table 2.

Character 21 - Closure of first gill slit. – Not unique to Thlasseleotrididae as discussed by Gill & Mooi
We have discarded the character from our matrix and analysis.

Characters 22-25 are really not autapomorphies as they occur in various taxa in other groups. Autapomorphies are defined as unique. Defining a character state as usually is not unique.
Character 22. Blind eleotrids, gobiids and oxudercids are known. Lack of eyes – not particularly good character. Lack of eye relates to living in caves. Probably not phylogenetically important. Most caves are far too recent to affect long term evolution.
Character 23. Not really polarized. Authors are in effect saying every character state that doesn’t agree with the presumed specialisation is the primitive condition. Also based on assumption that everything has 6 dorsal spines, which doesn’t work for temperate gobioids.
Character 24 over 100 new world species have 3(221110). Also as for 23.
About half of eleotrine genera have 3(1221), but Tateurndina3(121111) or 3(122101), Allomogurnda, Gobiomorphus and Mogurnda highly variable within a species and other genera with 7 spines.
Character 25 – “amblyopine” often have 3(1221), Rhinogobius, Tridentiger and some European genera 3(22110), supposedly an apomorphy for gobiids. Many north Pacific species have various patterns such as 122010, 12111010, etc. Character in table is missing something – should be 3(12210). Also as for 23.
As mentioned above, we have now removed all pterygiophore formulas from the matrix and phylogenetic considerations.

Hoese and Gill did not fully resolve the phylogeny and placement of dwarf gobioids, such as Kribia, Thalasseleotris, Grahamichthys, Tateurndina and dwarf “Oxyeleotris”, which did not have clear association with character states in other genera.
We have included the available information as much as possible

Authors should point out that gobioids are highly variable in most characters and discuss implication of that variation and how it could affect their analyses. Much of the variation is discussed in papers cited by the authors.
We present now two phylogenetic analyses based on maximum parsimony, one includes the intra-family variation of the interneural gap and dorsal postcleithrum (tree shown in Fig 11A), one excludes it (tree shown in Fig 11B). The topology of the trees is the same, but bootstrap support >50% for the node leading to the †Pirskeniidae + Thalasseleotrididae + Oxudercidae + Gobiidae is only present the latter tree (shown in Fig 11B).

Authors also might want to comment of Carlomonnius, a supposedly gobioid from Eocene of Italy.
We mention it now in the Introduction.

Reviewer #2: Thank you for the opportunity to review, please can you look at the attached PDF document with all of my detailed comments for this review added, this was a complex paper to review.
Many Thanks

**Reviewer #2:**

l. 42: Absence of evidence is NOT evidence of absence!
We have rephrased this sentence

l. 53: So you are saying that all five authors here agree on the phylogeny as presented in Figure 1? That would surprise me.
The cited authors are Agorreta et al. 2013, Hoese & Gill 1993, Miller 1973, Thacker 2009; Thacker et al. 2015. We have now deleted the hint to Fig. 1 from the sentence because we did not want to express that all cited authors agree on the phylogeny presented in Fig. 1. We wanted to give credit to Miller 1973 and Hoese & Gill 1993 because of their important works on the Rhyacichthyidae (by Miller) and Odontobutidae (by Hoese & Gill), and to the others because of their important works on gobioid phylogeny.

l. 54: I think you should substitute "The latter two..." or "These two..." for "They..." to remove all ambiguity as to what "They" refers to.
Done. We use now "These two.."

l. 73: Replace with "However, it was later...."
Done.

l. 77: Replace with "...become established in...". The presence of one or two species in a very limited area of the Czech Republic can scarcely be called a "conquest of European inland waters"!!
Done.

l. 148: delete "the name-giving genus"
Done.

l. 181: Change to "...can in principal..."
Done.

l. 188: Surely these are independant variables? So there are two characters here: 1) the number of branchiostegal rays (variable within gobioids) and 2) the last ray expanded (a gobioiod synapomorphy). So # 1 is informative, # 2 is irrrelevant.
As our focus is †*Pirksenius*, it is necessary to consider the characters in combination. Without that information, †*Pirksenius* would have the same character state (seven br rays) like the 'outgroup'.

l. 198: I think this section should be moved to the front of "Phylogenetic analysis", especially since it deals with ordering and weighting. I had asked several questions about that on the preceding pages only to find the answers here.
Done.

l. 241. OK, so you have an "L"-shaped palatine and a "T"-shaped palatine. So what are you calling the shape in Rhyacichthys? Table 2 says "inconstant" - not sure what that means, especially with respect to Rhyacichthys. The description you give above seems to be more or less T-shaped, or at the least state 2 (not exactly L-shaped).
We have improved the description, please see also revised Table 2 (character 14).

l. 282. Change to "...11672**. H**olotype NMP PC 2769..."Otherwise it looks like you are saying that the holotype is a "New specimen".
Done.

l. 442. Can you see whether there is the typical gobioid 'cleithral notch"?
Yes, it is there, we mention it now in the chapter "Paired fins and their skeleton" of †*P. diatomaceus*.

l. 449 ".. and appears more feasible" –
the sentence was rephrased to explain better what we mean.

l. 483. Replace with "discernable".
Done.

l. 538, 539: I would delete this sentence, and the next one. Do you really believe that presence/absence of anything is tied to a given level in the totally arbitrary and subjective field of higher classification? I doubt whether Doug would maintain those statements today.
OK, we deleted both sentences.

l. 557: Replace with "...of that species."
Done.

l. 584: I don't view that as "small". The 105 species of gobies I work with are 2.5 cms maximum, and the 120+ species od Eviota are even smaller. So 6 cms for a goby is pretty big to me.
OK, we changed "small fish" to "medium-sized gobioid fish".

l. 588: I don't think you can call this part of a "diagnosis" - suggest you delete it.
Done.

l. 614: Yes it does. But as an autapomorphy, it is of no help in establishing the phylogenetic relationships, and thus the appropriate hierarchical level, of the taxon.
We added this.

l. 617: Run on: "...process (Fig. 6a) **and it is probable that** †Pirskenius..."
Done.

l. 621: I think you should talk about them here - it seems a bit lazy not to!!
Done.

l. 624: Same comment as on l. 584:
OK, we changed "small fish" to "medium-sized gobioid fish".

l. 634: Here is where I have a HUGE problem. The resulting tree you give (Fig. 9) is fully resolved, despite the fact that there is no evidence given for two purported sister group relationships: (Thalasseleotrididae + Pirskeniidae; and Milyeringiidae + the rest of the gobioids to the exclusion of the Butidae). Those nodes collapse for lack of any evidence into two trichotomies (see my interpretation immediately after your Figure 9. As far as I know, there are only two ways a maximum parsimony analysis could be corrupted to give resolution where no supporting evidence exists. 1) It could, just like Maximum Liklihood, make a totally arbitrary decision among the three possibilities (Swofford told me this is what it does when I asked some decades ago). Or, b (and more likely) Goloboff has programmed it to make a bunch of predictions based on extraneous factors such as you listed here in line 209. Frankly, that kind of use of statistics is phenetics - not phylogenetics (very similar to the manipulations common in several programs to analyze supposed cladistic biogeographic data). I view such results as no better than random, and just as uninformative. Certainly, I do not believe these techniques should be used to generate cladograms that are then going to be used for interpretive predictions.

We agree with the comment on Figure 9, the Figure has been corrected and improved (now Fig 11) based on new analyses that take into account the intra-family variation of some characters, as recommended by Reviewer 1. Furthermore, we tested our phylogenetic interpretation making use of both TNT and PAUP*. The resulting trees are almost identical, but PAUP* provided a slightly better resolution and higher bootstrap values compared to TNT (see Fig. 11A-B of the revised version vs. S1 Fig A-B).

l. 647: Only if you are a pheneticist!! To a cladist it is simply a plesiomorphic character state at the level of analysis, and tells you nothing of the phylogenetic relationships.

The comment refers to the condition of "the penultimate branchiostegal ray [that] articulates at the anterior ceratohyal, but near to the gap that separates this part of the bone from the posterior ceratohyal". and the following sentence: "Such a condition is also known for the Odontobutidae [4], which could indicate a placement of †Pirskeniidae close to the Odontobutidae." It is true that this character state is plesiomorphic in the Odontobutidae with respect to Eleotridae, Butidae and Gobiidae, but with regard to the Rhyacichthyidae (as explained in the preceding sentence, l. 642) it is derived.

l. 647: This list is incomplete, and should be completed by adding Thalasseleotrididae and Milyeringiidae. We have replaced "Eleotridae, Butidae and Gobiidae" by "All other families".

l. 650: You list 3 - radial, scapula, and lack of transforming ctenii. We corrected "two" to "three".

l. 656: This statement will lead you into a quagmire from which I do not think there is any logical escape! Ok, the sentence has been changed to "Only with respect to the exact position of the penultimate branchiostegal, †Pirskeniidae is set apart from the remaining families, as indicated in Figure 8A."

l. 671-675: This part was apparently not clear, and unfortunately we did a mistake as we wrongly referred to character 1 (correct is character 8). We have rephrased the respective sentences.

l. 685: See extensive comment and protest on p. 26.

Please see response above.

l. 689: I think this is a very sad choice. This tree, using new and essentially untested software, is sharply different from the trees presented in Figs. 1 and 8 a, b. WHY???

Because our tree takes only into account morphological characters. We have rephrased this to explain it better.

l. 690: Replace: "...as **sequential** outgroups..." Not necessary any more, as Rhyacichthyidae and Odontobutidae form a sister relationship in the new tree.

l. 695: I think you need to look closely at the analysis and report your findings here. There has to be at least one reason for the analysis to place it as the sister group of the Thalasseleotrididae if it is an MP analysis. From the figure, there is no character given, so this is in fact more like a Maximum Likelihood analysis. ML will choose (randomly) one resolved tree when there is an unresolved tri- or poly-chotomy. That seems to be the case here and, unless you can find a synapomorphy shared by Th. and Pirs., you should alter Fig. 9 to reflect a trichotomy of these two with the Oxudercidae + Gobiidae. See alternate tree to Fig. 9.

The new tree (Fig. 11) takes into account these concerns.

The conclusion was shortened as proposed by Reviewer 2

6. PLOS authors have the option to publish the peer review history of their article ([what does this mean?](https://journals.plos.org/plosone/s/editorial-and-peer-review-process#loc-peer-review-history)). If published, this will include your full peer review and any attached files.


If you choose “no”, your identity will remain anonymous but your review may still be made public.


**Do you want your identity to be public for this peer review?** For information about this choice, including consent withdrawal, please see our [Privacy Policy](https://www.plos.org/privacy-policy).

Reviewer #1: Yes: Douglass Hoese

Reviewer #2: No


[NOTE: If reviewer comments were submitted as an attachment file, they will be attached to this email and accessible via the submission site. Please log into your account, locate the manuscript record, and check for the action link "View Attachments". If this link does not appear, there are no attachment files.]

While revising your submission, please upload your figure files to the Preflight Analysis and Conversion Engine (PACE) digital diagnostic tool, <https://pacev2.apexcovantage.com/>. PACE helps ensure that figures meet PLOS requirements. To use PACE, you must first register as a user. Registration is free. Then, login and navigate to the UPLOAD tab, where you will find detailed instructions on how to use the tool. If you encounter any issues or have any questions when using PACE, please email PLOS at [figures@plos.org](mailto:figures@plos.org). Please note that Supporting Information files do not need this step.

*In compliance with data protection regulations, you may request that we remove your personal registration details at any time.* [*(Remove my information/details)*](https://www.editorialmanager.com/pone/login.asp?a=r)*. Please contact the publication office if you have any questions.*
